# Optimization Strategy for Generating Gene-edited Tibet Minipigs by Synchronized Oestrus and Cytoplasmic Microinjection

**DOI:** 10.7150/ijbs.35930

**Published:** 2019-10-15

**Authors:** Bangzhu Chen, Peng Gu, Junshuang Jia, Wei Liu, Yumin Liu, Wen Liu, Tao Xu, Xiaolin Lin, Taoyan Lin, Yu Liu, Hengwei Chen, Mingchen Xu, Jin Yuan, Jianing Zhang, Yinghui Zhang, Dong Xiao, Weiwang Gu

**Affiliations:** 1 School of Basic Medical Sciences, Southern Medical University, Guangzhou 510515, China; 2 Institute of Comparative Medicine & Laboratory Animal Center, Southern Medical University, Guangzhou 510515, China; 3 Songshan Lake Pearl Laboratory Animal Sci. & Tech. Co., Ltd., Dongguan 523808, China; 4 Guangdong Provincial Key Laboratory of Cancer Immunotherapy Research and Guangzhou Key Laboratory of Tumor Immunology Research, Cancer Research Institute, Southern Medical University, Guangzhou 510515, China; 5 School of Biotechnology and Health Sciences, Wuyi University, Jiangmen 529020, China

**Keywords:** Tibet minipig, cytoplasmic injection, gene editing, oestrus synchronization, CRISPR/Cas9

## Abstract

The Tibet minipig is a rare highland pig breed worldwide and has many applications in biomedical and agricultural research. However, Tibet minipigs are not like domesticated pigs in that their ovulation number is low, which is unfavourable for the collection of zygotes. Partly for this reason, few studies have reported the successful generation of genetically modified Tibet minipigs by zygote injection. To address this issue, we described an efficient way to generate gene-edited Tibet minipigs, the major elements of which include the utilization of synchronized oestrus instead of superovulation to obtain zygotes, optimization of the preparation strategy, and co-injection of clustered regularly interspaced short palindromic repeat sequences associated protein 9 (Cas9) mRNA and single-guide RNAs (sgRNAs) into the cytoplasm of zygotes. We successfully obtained allelic *TYR* gene knockout (*TYR*^-/-^) Tibet minipigs with a typical albino phenotype (i.e., red-coloured eyes with light pink-tinted irises and no pigmentation in the skin and hair) as well as *TYR*^-/-^*IL2RG*^-/-^ and *TYR*^-/-^*RAG1*^-/-^ Tibet minipigs with typical phenotypes of albinism and immunodeficiency, which was characterized by thymic atrophy and abnormal immunocyte proportions. The overall gene editing efficiency was 75% for the *TYR* single gene knockout, while for *TYR-IL2RG* and *TYR-RAG1* dual gene editing, the values were 25% and 75%, respectively. No detectable off-target mutations were observed. By intercrossing F_0_ generation minipigs, targeted genetic mutations can also be transmitted to gene-edited minipigs' offspring through germ line transmission. This study is a valuable exploration for the efficient generation of gene-edited Tibet minipigs with medical research value in the future.

## Introduction

As a rare highland pig breed worldwide, Tibet minipigs originally inhabited the highland region of Tibet, China^1^. They are small in size, they are characterized by good adaptability to harsh environmental conditions and superior disease-resistance capability^2^, and they have many applications in biomedical and agricultural research. Unlike domesticated pigs, the related reproductive genes of Tibet minipigs have successfully evolved to produce offspring under harsh conditions at high altitudes^3^. On the other hand, Tibet minipigs produce heavier and stronger piglets by sacrificing their litter size (four to eight piglets per litter)^3^. We can obtain only a limited number of embryos from Tibet minipigs in natural oestrous, which severely limits the generation of gene-edited Tibet minipigs through embryo injection. With progress in gene editing technology, especially the emergence of CRISPR gene editing tools^4^, ^5^, researchers began to adopt a zygote cytoplasmic injection method to generate genetically modified animals^6^-^10^. Although this method is simpler and more technically convenient than somatic cell nuclear transfer (SCNT)^11^, there is still no report on the successful generation of genetically modified Tibet minipigs by the zygote cytoplasmic injection method. Here, we aimed to explore a feasible way to generate gene-edited Tibet minipigs by embryo cytoplasmic microinjection of the CRISPR/Cas9 system.

We did not choose the traditional superovulation method to obtain Tibet minipig zygotes due to its less satisfactory applicability in Tibet minipigs. Instead, we adopted another simple and effective strategy: altrenogest has been proven to be efficient in synchronizing oestrus in pigs^12^, ^13^. Oestrus synchronization by feeding altrenogest to Tibet minipigs makes them enter into oestrus at the same time. In this way, the donor Tibet minipigs can mate on the same day, and enough zygotes are gathered on the next day. The tyrosinase gene (*TYR*) in Tibet minipigs, encoding tyrosinase, which is responsible for the biochemical synthesis of melanin^14^, was initially chosen as the target gene. Mutation of *TYR* in animals often leads to inhibition of the melanin synthesis pathway, resulting in partial pigment loss or albinism^15^. Therefore, chimeras composed of *TYR* mutant and wild-type (WT) melanocyte cells can be evaluated by visual examination^15^, ^16^. The CRISPR/Cas9 system can also be used to efficiently knock out multiple genes in embryos^8^. As such, we also aimed to target two genes in the same zygotes. We targeted the *TYR* gene as well as the interleukin 2 receptor gamma gene (*IL2RG*) or recombinant activating gene 1 (*RAG1*). *IL2RG* maps to the X chromosome and encodes a common gamma chain protein, which is a subunit of various interleukin receptors involved in the immune system^17^. Pigs lacking functional *IL2RG* show symptoms of immune deficiency, including thymic atrophy, decreased or absent T lymphocytes and natural killer (NK) cells, and attenuation of B cell function^18^-^21^. The RAG1 protein, encoded by the *RAG1* gene, is involved in V(D)J recombination (recombination of numerous variable (V), diversity (D), and joining (J) gene segments) of the immunoglobulin light and heavy chain gene loci, which is crucial to the early development of T lymphocytes and B lymphocytes^22^, ^23^. *RAG1* deficiency also causes immunodeficiency in pigs^24^, ^25^.

In this context, we describe an efficient way to prepare gene-edited Tibet minipigs by a combination of synchronized oestrus and zygote cytoplasmic microinjection. We successfully generated *TYR*-KO, *IL2RG*-KO, and *RAG1*-KO Tibet minipigs, which are phenotypically characterized by albinism and/or immunodeficiency. Technically, we have also optimized the generation process of genetically modified pigs. This study is a valuable exploration to efficiently generate genetically modified Tibet minipigs for further medical research purposes.

## Materials and Methods

### Animals

The Tibet minipigs used in this study were raised in the Laboratory Animal Center, Southern Medical University (Guangzhou, China). All animal protocols were approved by the Institutional Animal Care and Use Committee (IACUC) at the Institute of the Laboratory Animal Center, Southern Medical University (Animal Welfare Assurance, L2016088). All surgery was performed under anaesthesia, and all efforts were made to minimize animal suffering.

### Design and construction of the CRISPR/Cas9 system

The sgRNA cloning vector was pGL3-U6-gRNA-PGK-puromycin (Addgene plasmid 51133), and the Cas9 expressing plasmid was pST1374-NLS-flag-linker-Cas9 (Addgene plasmid 44758). The sgRNAs targeting the Tibet minipig *TYR*, *IL2RG*, and *RAG1* genes were designed online (http://crispor.tefor.net/). Generation of sgRNAs was performed as previously described^26^. Briefly, sgRNA oligonucleotide sequences complementary to the *TYR*, *IL2RG*, and *RAG1* genes (**Table S1**) were annealed and cloned into the BsaI site of pGL3-U6-sgRNA-PGK-Puro. The T7 promoter was then added to the sgRNA template by PCR amplification of sgRNA expression plasmids using the primers listed in** Table S1**. T7-sgRNA PCR products were purified and used as templates for synthesis of sgRNAs using the MEGAshortscript T7 Transcription Kit (AM1354, Life Technologies, USA). Plasmid DNA of pST1374-NLS-flag-linker-Cas9 was completely digested using the *Age* I restriction endonuclease, purified, and then used as a template for in vitro synthesis of Cas9 mRNA by the mMESSAGE mMACHINE T7 ULTRA Kit (AM1345, Life Technologies, USA). Both sgRNAs and Cas9 mRNA were purified by phenol:chloroform extraction and ethanol precipitation.

### Synchronization of oestrus in Tibet minipigs

Healthy pubertal Tibet minipig gilts (approximately 7-9 months of age, 30-40 kg body weight) were selected as donors for embryo collection. Healthy Tibet minipig sows (over 12 months old) were selected as surrogates or nanny pigs. The chemical used for oestrus synchronization was prepared by dissolving 2 g altrenogest (Altrenogest; Beijing Keyifeng Biotech Develop. Co., Ltd., China) in 20 ml ethanol, which was then emulsified by mixing with 2 ml polysorbate-20, and vegetable oil was finally added to reach a volume of 400 ml. A 5 mg/ml altrenogest emulsion was obtained and fully shaken before use. All donor pigs, surrogate pigs, and nanny pigs were simultaneously treated with oestrus synchronization. Each pig was fed 20 mg altrenogest every morning once a day for 18 days. Most of the pigs entered into oestrus within 5-8 days after drug withdrawal.

### Observation of oestrus

When sows in full oestrus meet boars, they appear to be motionless. To examine whether the best oestrous state was reached, crystal violet staining of vaginal smears was performed as follows. First, a wet cotton swab was gently inserted into the sow's vagina and rotated several times. The cotton swab was removed, and a slide was coated with the cells and allowed to dry naturally. Then, the sample slides were dehydrated with methanol for 30 s, dried with methanol, and stained with crystal violet for 1 min. Finally, the slides were rinsed three times with clear water and dried again prior to examination under an optical microscope.

### In vivo collection of porcine zygotes

Zygotes were collected surgically between 16 and 18 h after mating. Prior to surgery, the animals were fasted for 12 h. Anaesthesia was induced by intravenous injection of pentobarbital sodium at a dose of 30-45 mg/kg. The ovary and uterus were exposed by lower abdominal surgery and flushed with Dulbecco's phosphate-buffered saline solution (DPBS; 21-031-CVR, Corning, USA) containing 1% bovine serum albumin. Zygotes were collected under a stereomicroscope.

### Cytoplasmic microinjection of Cas9 mRNA and sgRNAs

The concentrations of injected RNAs were 100 ng/µl (Cas9 mRNA) and 50 ng/µl (each sgRNA). Microinjections were executed using a microinjection apparatus (TransferMan NK2, Eppendorf, Germany). Cas9 mRNA plus sgRNAs were microinjected into the cytoplasm of 1-cell stage or 2-cell stage embryos. After microinjection, embryos were cultured in PZM3 medium before being transplanted to surrogates 1 h later.

### Embryo transplantation

Embryo transplantation was performed through surgery. After surrogate sows in oestrus were anaesthetized, a small incision was made in the groin area to expose the ovaries and fallopian tubes. Embryos were slowly injected into a fallopian tube, followed by suturing the wound. Nineteen to twenty-one days after transplantation, pregnancy test kits (Q/CPWHS 022-2000, Beijing Wanhua Bioengineering Co., Ltd., China) were used to detect pregnancy.

### Detection of target gene mutations

Mutant pig genotypes were determined by PCR and Sanger sequencing. Genomic DNA was extracted from the pig skin and semen. Boar semen samples were obtained via the gloved hand method and diluted in phosphate-buffered saline. The primers for the target gene loci are listed in **Table S2**. The PCR conditions were as follows: pre-denaturation at 94°C for 5 min, followed by 35 amplification cycles at 94°C for 30 s, 62°C for 30 s, and 72°C for 40 s, with a final extension at 72°C for 5 min. The PCR products were purified using a gel extraction purification kit and then cloned into the pMD-19-T vector (6013, Takara) following the manufacturer's instructions. Recombinant colonies selected on plates were picked from each transformation and screened by PCR using *TYR*, *IL2RG*, or *RAG1* gene-specific primers, and then Sanger sequencing was applied to detect mutations. At least 10 clones were sequenced from each pig.

### Off-target analysis

Potential CRISPR/Cas9 off-target sites (OTSs) in the pig genome were predicted using the CRISPR design tool (http://crispor.tefor.net/). Among them, ten potential OTSs for each sgRNA were selected (**Table S3**). All potential OTSs were amplified by PCR, and the PCR products were sequenced to confirm whether off-target effects had occurred. The primer pairs used to amplify candidate OTSs are listed in **Table S3**.

### Ammoniacal silver-nuclear fast red staining of Tibet minipig tissues to evaluate melanin distribution

Skin sections and eye sections were dewaxed and washed in distilled water. After rewashing, they were placed in silver ammonia hydroxide solution and incubated in a dark place at room temperature for 18 h. Subsequently, the sections were quickly washed in distilled water and then immersed in 0.2% chlorinated gold solution dye for 2 min. Following rewashing in distilled water, the sections were fixed in 5% sodium thiosulphate solution for 3 min. The sections were then rinsed in running water for 10 min and redyed for 5 min with nuclear fast red. Section preparation was completed by dehydration, hyalinization, and sealing.

### Flow cytometric analysis of immune cell populations

Peripheral blood mononuclear cells (PBMCs) were isolated from whole blood samples from *RAG1*-KO and age-matched control Tibet minipigs. To identify porcine CD4^+^CD3^+^ T, CD8^+^CD3^+^ T, CD45RA^+^CD3^-^ B, and CD16^+^CD3^-^ NK cells, specific fluorophore-conjugated primary antibodies (pAbs) were used, including Alexa Fluor 647-conjugated mouse anti-pig CD3e (561476, BD Bioscience Pharmingen, USA), FITC-conjugated mouse anti-pig CD4a (559585, BD Bioscience Pharmingen, USA), PE-conjugated mouse anti-pig CD8a (559584, BD Bioscience Pharmingen, USA), FITC-conjugated mouse anti-pig CD16 (G7, AbDSerotec, USA), and PE-conjugated mouse anti-pig CD45RA (MIL13, AbDSerotec, USA). A total of 5 × 10^4^ PBMCs were incubated with the indicated antibodies for 30 min at 4°C and washed twice with PBS. At least 10,000 cells were analysed per run. Samples were analysed using a BD LSRFortessa cell analyser (647794, BD Bioscience, USA), and data were processed using BD FACSDiva software.

### Histological analysis of immunodeficient minipigs

The spleens of *IL2RG*-KO minipigs, *RAG*-KO minipigs and age-matched wild-type minipigs were first fixed with 4% paraformaldehyde and then cut into paraffin sections. The sections were stained with H&E for histological analysis and analysed through immunohistochemistry (IHC). In IHC, rabbit anti-CD3 pAb (GB11014, Servicebio, China) was used to identify T lymphocytes, and rabbit anti-CD19 pAb (GB11061-1, Servicebio, China) was used to identify B lymphocytes. Rabbit anti-CD11b pAb (GB11058, Servicebio, China) was used to identify macrophages, neutrophil granulocytes and NK cells.

### Quantification and statistical analysis

To quantify the results of IHC, three sections were selected for each group. Six non-overlapping fields (10-fold objective) were randomly selected for each section and scanned by Toup View system (Beijing Top View Technology Co., Ltd, China). The positive stained cells in each field were counted artificially. The average numbers of positive stained cells per field (10-fold objective) were used for statistical analysis. Student's t-test was used for statistical analysis between every two groups. Data are expressed as mean ± SEM. Statistical analyses were performed with GraphPad Prism software (GraphPad Software, USA). The P value less than 0.05 was considered as statistically significant.

## Results

### Optimization of the synchronous oestrus strategy

**Figure 1:**
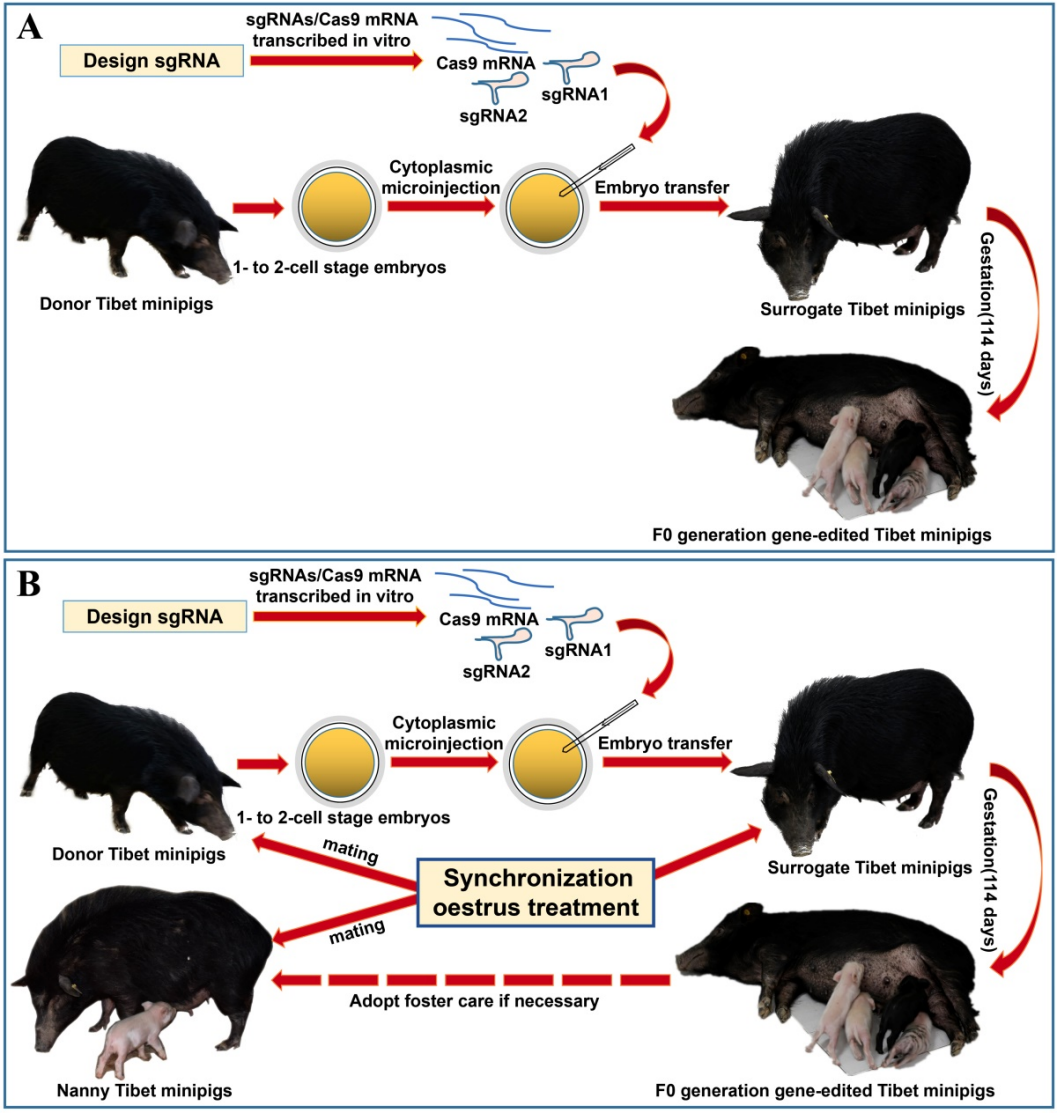
** Strategy for generating gene-edited Tibet Minipigs. A.** Conventional strategy for generating gene-edited Tibet Minipigs: wild-type Tibet minipigs in oestrus were selected as embryo donors. Zygotes were obtained by surgery, followed by microinjection of Cas9 mRNA plus sgRNAs into the zygote cytoplasm. Modified embryos were transplanted into surrogate gilts in natural oestrus, which delivered gene-edited piglets after normal gestation. **B.** Optimized strategy: oestrus was synchronized in wild-type Tibet minipigs, which were assigned as donor pigs, surrogate pigs and nanny pigs. Both donor pigs and nanny pigs mated at the same time. Zygotes were edited and transferred to the surrogate sows. Nanny pigs can breastfeed some F_0_ gene-modified piglets on behalf of surrogate sows, who may fail to fulfil their nurturing role, as experimentally observed. This approach significantly improved the survival rate of F_0_ generation pigs after birth.

Considering the limited source of zygotes and the surrogate sows' occasional failure to feed piglets, we devised the procedure for oestrus synchronization and introduced nanny pigs to optimize the conventional strategy of preparing gene-edited pigs by zygote injection (**Figure 1A**)^8^. Specifically, by using synchronized oestrus treatment instead of the superovulation method (**Figure 1B**), on average, 6 zygotes were obtained from each donor gilt, which effectively fulfilled the embryo quantity requirement (**Table 1**). To avoid the problem that F_0_ generation piglets cannot be raised by surrogate sows, nanny pigs that also underwent synchronized oestrus and mating processes were introduced during the preparation of gene-edited Tibet minipigs. This enabled the nanny pigs to give birth at a very close time point to the surrogate sows. If the surrogate sow could not nurse or raise the piglets, then the nanny pig filled its role (**Figure 1B**).

**Figure 2:**
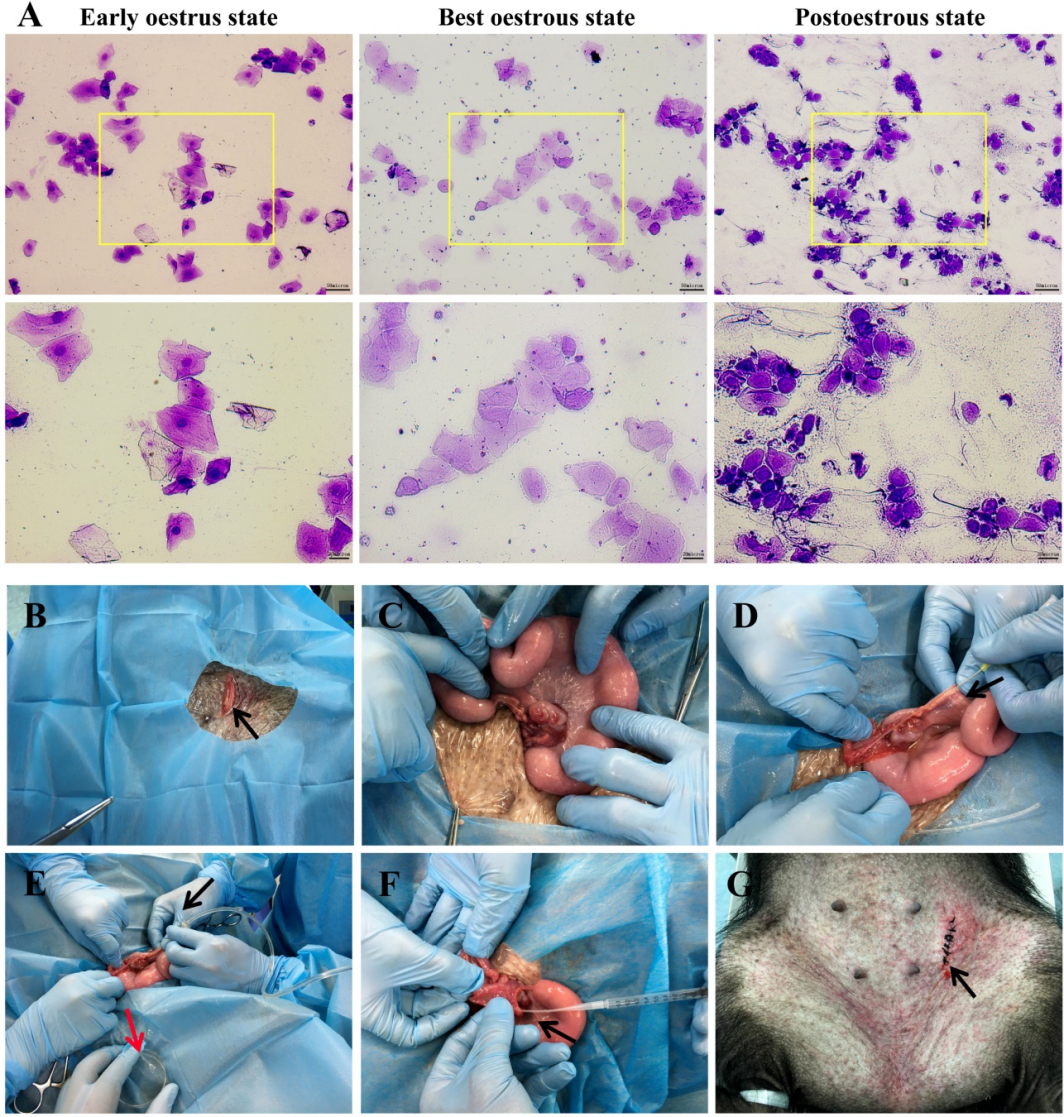
** Oestrus status check, zygote collection and embryo transplantation. A.** Estimation of oestrus status. Early oestrus state: there are many large polygonal keratinocytes with their nuclei clearly stained. Best oestrous state: large-diameter polygonal keratinocytes detected with shallow staining, wrinkled edges and nuclei disappeared. Postoestrous state: the number of large-diameter polygonal keratinocytes decreased, and small round epidermal cells reappeared with deeper staining. Neutrophils and filamentous viscous mucus could also be observed. Scale bars of low and high magnification images are 50 µm and 20 µm, respectively.** B-E.** Zygote collection. A small incision (black arrow) was opened in the groin area **(B)**, and then the ovaries and fallopian tubes were exposed **(C)**. After insertion of a needle (black arrow) into the isthmus of the fallopian tube **(D)**, the oviduct was flushed with 10 ml of prewarmed DPBS (black arrow), and simultaneously, a sterile plastic pipe was inserted into the infundibulum tubae uterinae to collect the flushing fluid into a Petri dish (red arrow) **(E)**. **F-G**. Embryo transplantation. An embryo transfer catheter was inserted from the opening of the fallopian tube (black arrow), and embryos were slowly injected into the fallopian tube **(F)**. Finally, the wound was sutured (black arrow) **(G)**.

The best oestrous state occurred within 5-7 days after altrenogest withdrawal, when most of the cells in the vaginal smears were large keratinized epidermal cells that contained no nucleus and displayed edge wrinkles (**Figure 2A, best oestrous state**). If donor sows mate prematurely (**Figure 2A, early oestrus state**), they may not ovulate the next day; if mating times are postponed too much (**Figure 2A, postoestrous state**), the donor sows will no longer remain in oestrus and will not mate. Embryo collection (**Figure 2B-E**) and embryo transfer (**Figure 2F-G**) were performed as described.

### Generation of Tibet minipigs with targeted gene mutations

**Figure 3:**
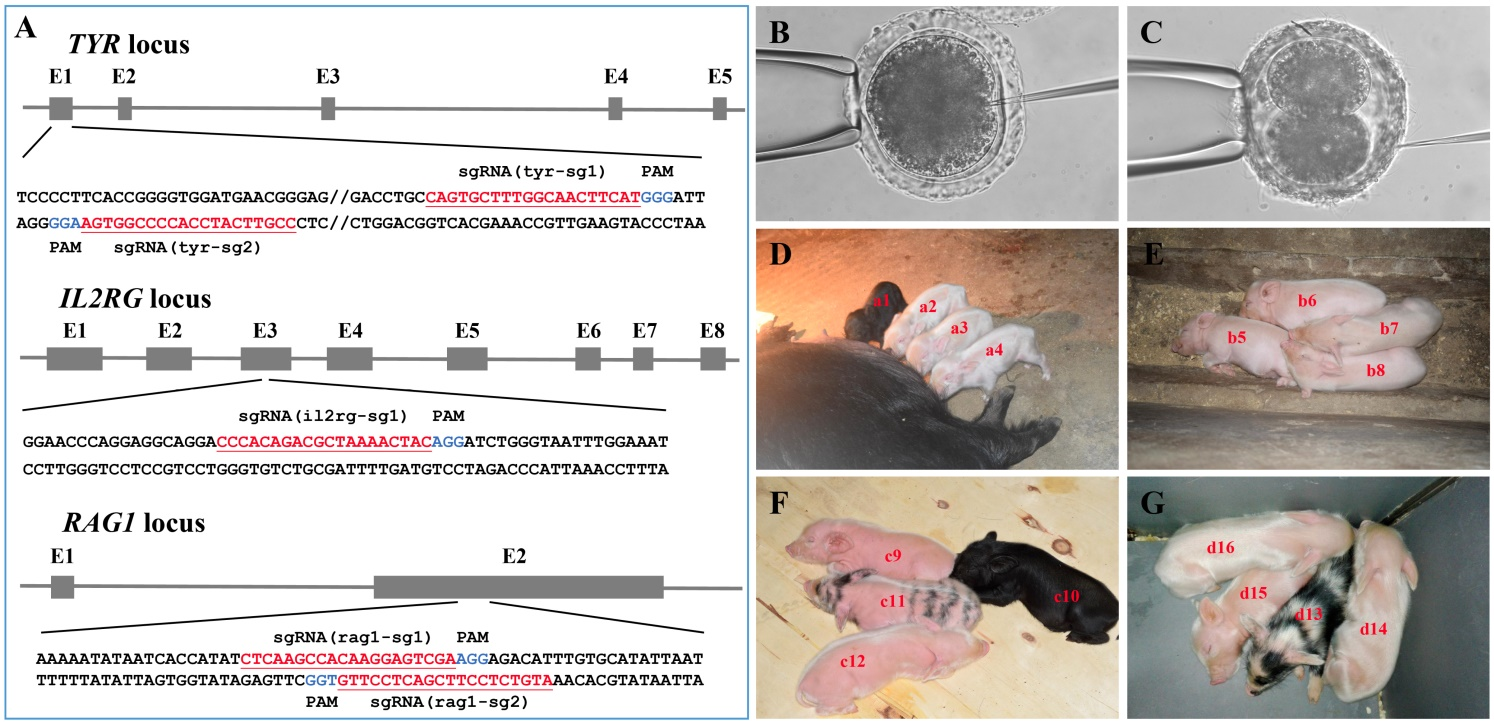
**Generation of gene-modified Tibet minipigs by Cas9 mRNA/sgRNAs cytoplasmic microinjection. A.** Schematic diagram of sgRNAs targeting *TYR*, *IL2RG*, and *RAG1* loci. The *TYR*, *IL2RG* or *RAG1* target site(s) were located in exons 1, 3 and 2, respectively. The sgRNA targeting sites are underlined and highlighted in red. Protospacer adjacent motifs (PAM) are indicated in blue. **B-C.** Schematic diagram of Cas9 mRNA plus sgRNA microinjection into the cytoplasm of 1-cell stage (**B**) or 2-cell stage (**C**) embryos.** D.** Surrogate A gave birth to four piglets (i.e., a1, a2, a3 and a4). **E.** Surrogate B gave birth to four piglets (i.e., b5, b6, b7 and b8). **F.** Surrogate C gave birth to four piglets (i.e., c9, c10, c11 and c12). **G.** Surrogate D gave birth to four piglets (i.e., d13, d14, d15, and d16).

As shown in **Figure 3A** and **Table S1**, multiple sgRNAs specifically targeting *TYR*, *IL2RG*, or *RAG1* were designed. A total of 74 embryos were injected with the Cas9 mRNA/sgRNA mixture and transplanted into seven surrogate sows in oestrus, and four pregnancies were established (referred to as surrogates A, B, C, and D) (**Table 2**). Embryos in which the *TYR* gene was to be edited were microinjected at the 1-cell stage (**Figure 3B**) and then transplanted into surrogates A and B, each of which gave birth to four piglets (**Table 2**). Dual gene-targeted (*TYR-IL2RG* or *TYR*-*RAG1*) embryos were microinjected at the 2-cell stage (**Figure 3C**) and then transplanted into surrogates C and D, respectively, each of which also gave birth to four piglets (**Table 2**). One reason for choosing 2-cell stage embryos in our experimental design was that *IL2RG* or *RAG1* gene knockout caused severe immunodeficiency in the animals, resulting in a short lifespan^18^, ^20^, ^24^, ^27^, while gene modification at the 2-cell stage embryo may generate chimeric animals, which can survive and pass on the mutated *RAG1* or *IL2RG* gene to their offspring.

As observed, all pregnant surrogates were delivered on 114 ± 1 days without a delay in delivery (**Table 2**). Among the 16 F_0_ generation piglets obtained, the male-to-female sex ratio was 9:7, and the percentage of births relative to embryos transferred ranged from 33.3% to 50% (**Table 2**). Twelve out of 16 F_0_ generation piglets (i.e., a2, a3, a4, b5, b6, b7, b8, c9, c12, d14, d15 and d16) were albino, except for 4 F_0_ generation piglets that were black (i.e., a1 and c10) or exhibited partial pigment loss (i.e., c11 and d13) (**Figure 3D-G, Table 3**). In addition, we obtained dual gene knockout piglets [i.e., c9 (*TYR*^-/-^*IL2RG*^-/-^), d14 (*TYR*^-/-^*RAG1*^-/-^) and d15 (*TYR*^-/-^*RAG1*^-/-^)] had phenotypes of both albinism and immunodeficiency, whereas some chimeras (i.e., piglet c12 carrying the *TYR* and *IL2RG* mutations) had the albino phenotype but did not exhibit immunodeficiency (**Table 3**).

### Genotype analysis of CRISPR/Cas9-induced targeted gene mutations in minipigs

To further characterize the CRISPR/Cas9-induced targeted gene mutations, genotype analysis was conducted by Sanger sequencing of samples from all F_0_ generation piglets (**Table 3**). The results indicated that targeted gene locus mutations were characterized by short indels of various lengths or a various number of base pair substitutions (**Tables S4-S6**). These changes in the DNA sequence usually resulted in truncated coding regions or coding frameshifts, thereby leading to *TYR*, *IL2RG*, or *RAG1* gene dysfunction.

Moreover, in some piglets, sequence-based genotype analysis revealed multiple allelic mutations of the target gene(s) (**Table S4, Table S5**), indicating that the piglets were chimeric, as shown in piglets a3, b8, c10, c11, and d13, which had *TYR* gene mutations (**Table S4**), and piglet c12, which had *IL2RG* mutations (**Table S5**). Chimeric piglets mainly developed from dual-gene targeted embryos, which were microinjected at the 2-cell stage.

Of the 16 F_0_ generation piglets, 12 had complete knockout of the *TYR* gene, representing a complete knockout efficiency of 75% (**Table 2**). Editing of 2-cell stage embryos using the CRISPR/Cas9 system (**Table 2**) generated three piglets (c9, d14, and d15) with complete double-gene knockouts (**Table 3, Tables S4-S6**). Piglet d13 had no immune abnormalities, most likely because the 9 bp deletion detected in its *RAG1* gene target did not cause a coding frameshift. The double-gene modification efficiencies for *TYR*-*IL2RG* and *TYR*-*RAG1* were 25% and 75%, respectively (**Table 2**). The genotype data corresponded well with the observed phenotypes of albinism and/or immunodeficiency observed in the F_0_ generation piglets (**Figure 3D-G** and **Table 3**).

### Further analysis of the phenotypes of* TYR*, *IL2RG*, and *RAG1* gene-targeted Tibet minipigs

**Figure 4:**
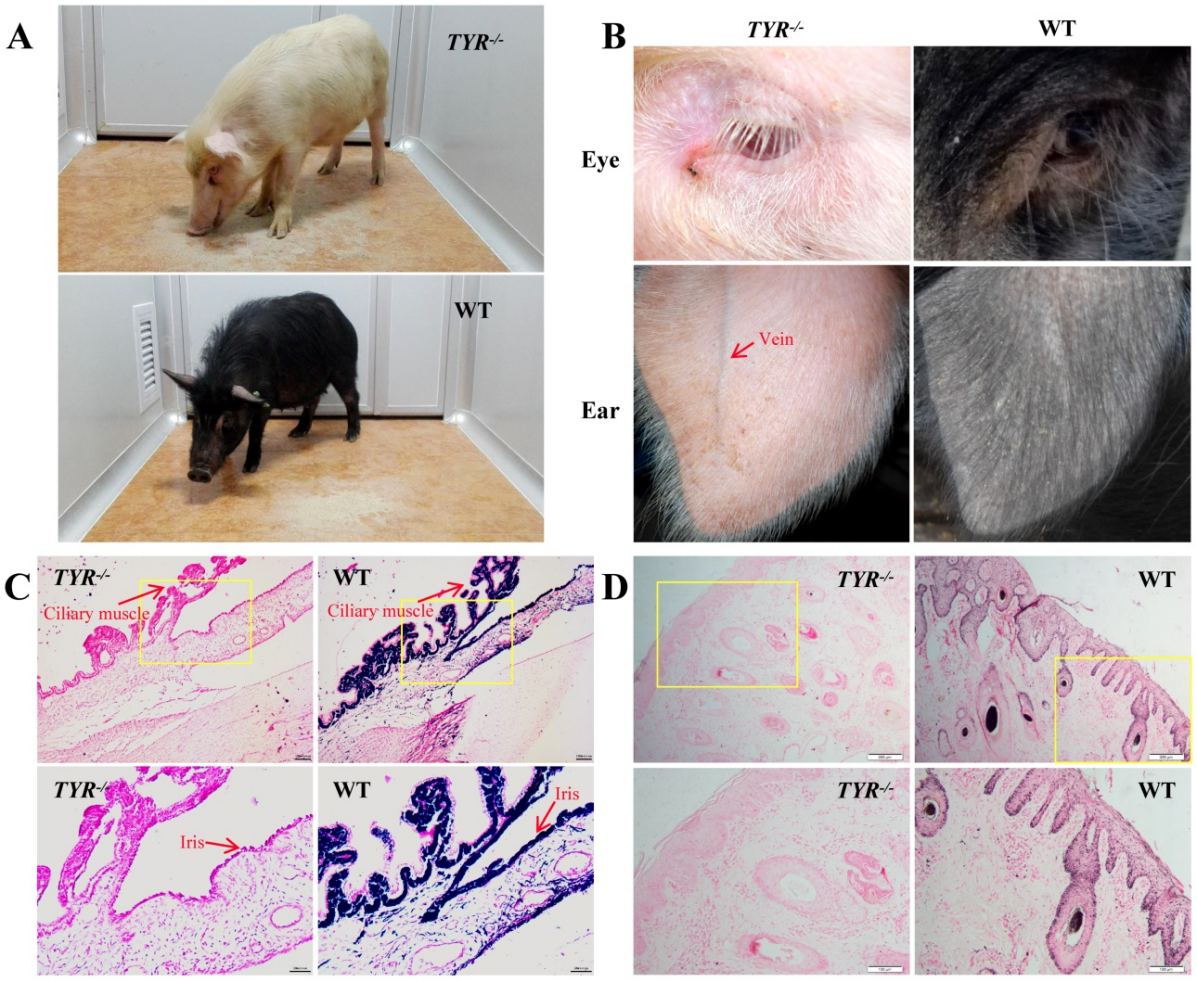
***TYR*-KO Tibet minipigs exhibit an albino phenotype. A.** White and black hair colour contrast between adult *TYR*-KO and WT Tibet minipigs.** B.** Comparison of eye and ear tissues from *TYR*-KO and WT Tibet minipigs. **C.** Ammoniacal silver-nuclear fast red staining of the ciliary muscle and iris of WT and *TYR*-KO Tibet minipigs. Scale bars of low and high magnification images are 100 µm and 50 µm, respectively. **D.** Ammoniacal silver-nuclear fast red staining of the ear skin of WT and *TYR*-KO Tibet minipigs. Scale bars of low and high magnification images are 200 µm and 100 µm, respectively.

Pigmentation levels in the skin and hair are a good marker of whether *TYR* gene function is completely knocked out in F_0_ generation piglets^16^. We observed some chimeric piglets with partial pigment loss, suggesting incomplete knockout of the *TYR* gene (**Figure 3F, G**; piglets with black and white hair). Piglets (**Figure 3D-G**; piglets with white hair colour) and adult mutant Tibet minipigs (**Figure 4A**) with complete *TYR* gene knockout (*TYR*^-/-^) displayed a full-body albino phenotype, as supported by sequence-based genotype data (**Table S4**).

Phenotypically, *TYR*-KO Tibet minipigs exhibited the typical features of albinism (**Figure 4A**), including red-coloured eyes with light pink-tinted irises (**Figure 4B, C**) and no pigmentation in the skin (**Figure 4B, D**), in contrast to the dark irises and black skin of wild-type Tibet minipigs (**Figure 4A-D**). There was no comparable melanin distribution in the ciliary muscle and irises of *TYR*-KO Tibet minipigs, whereas melanin was distributed in the basal layer of the epidermis and detected in hair strand cross-sections in the WT Tibet minipig (**Figure 4C, D**). Visually, semi-transparency of the skin makes observation of the subcutaneous blood vessels in the ears of albino Tibet minipigs more convenient than that in wild-type Tibet minipigs, facilitating intravenous injection (**Figure 4B**).

**Figure 5:**
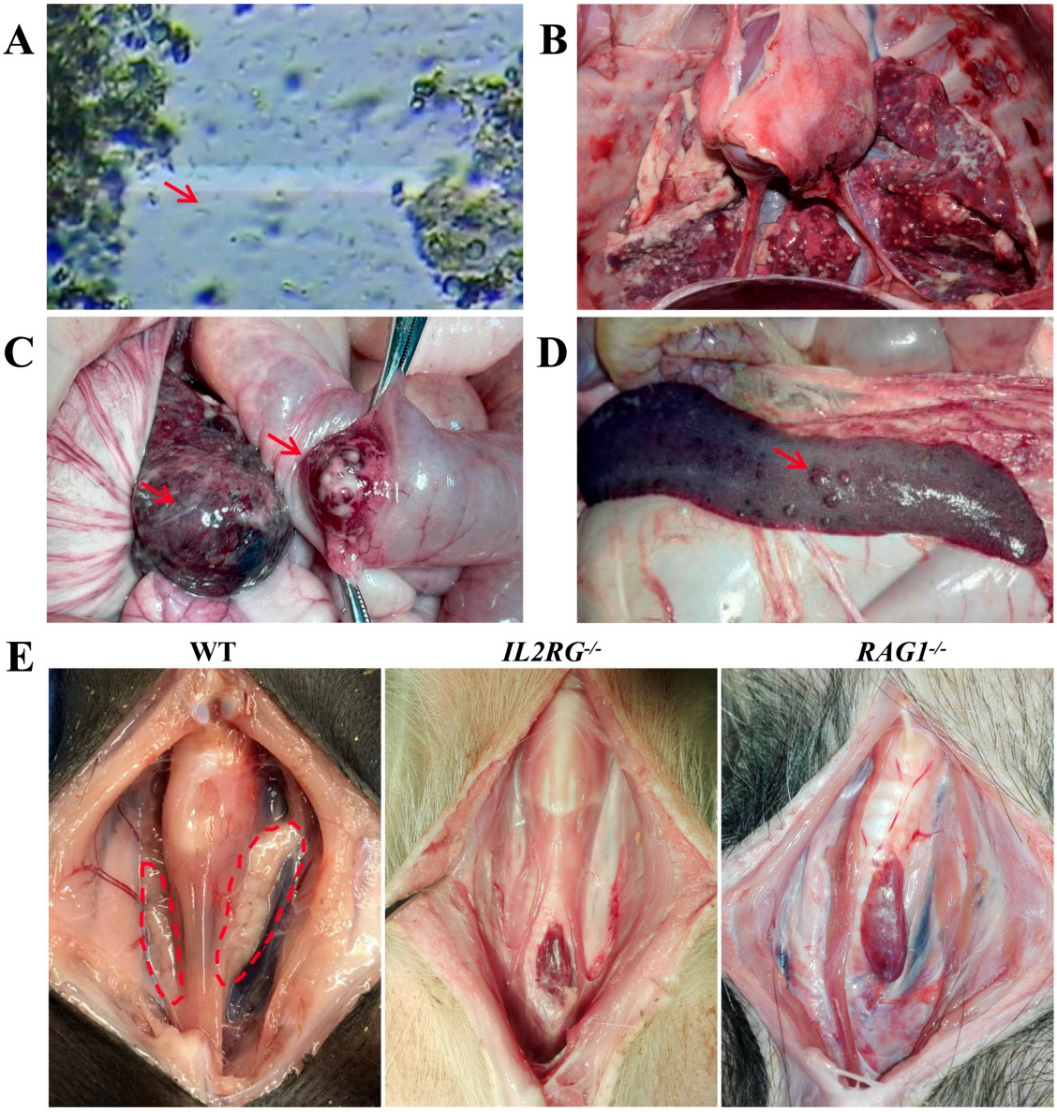
** Immunodeficiency symptoms and signs of *IL2RG*-KO and *RAG1*-KO Tibet minipigs. A.** Faecal microscopic examination of piglet c9 *(IL2RG*^-/-^) revealing flagellates (red arrowhead). **B.** Autopsy of piglet c9 (*IL2RG*^-/-^) revealed severe lung infection. **C.** Autopsy of piglet d14 (*RAG1*^-/-^) showing colon cancer (red arrowhead). **D.** Autopsy of piglet d14 (*RAG1*^-/-^) showing metastatic lesions in the spleen (red arrowhead). **E.** Absence of the thymus was observed in piglets c9 (*IL2RG*^-/-^) and d13 (*RAG1*^-/-^). The dotted red circle indicates the normal physiological site of the thymus.

To characterize the immunodeficient phenotype of mutant Tibet minipigs, piglet c9 was selected because genotype analysis indicated that it was an *IL2RG-*KO piglet (*IL2RG*^-/-^) (**Table S5**). To evaluate its immunodeficient features, the *IL2RG*-KO piglet was raised by breastfeeding in a clean environment. The piglet grew well in the early stage of its life; however, it later began to succumb to common bacterial infections of the digestive tract, which were then aggravated by multiple additional infections, such as flagellates (**Figure 5A**) and pneumonia (**Figure 5B**). Piglet c9 survived for only 132 days; autopsy detected the absence of a thymus (**Figure 5E**). Piglets d13, d14, d15, and d16 were *RAG1*-modified (**Table S6**). Unexpectedly, piglet d16 did not exhibit any symptoms of immunodeficiency, although genotype data revealed a 9 bp deletion in the *RAG1* gene. Other piglets died at 3-4.5 months of age. Piglet d14 survived only 95 days, and postmortem examination revealed colon cancer (**Figure 5C**) and metastatic lesions in the spleen (**Figure 5D**). Autopsy of piglet d13 revealed that its thymus was almost completely atrophied (**Figure 5E**).

**Figure 6:**
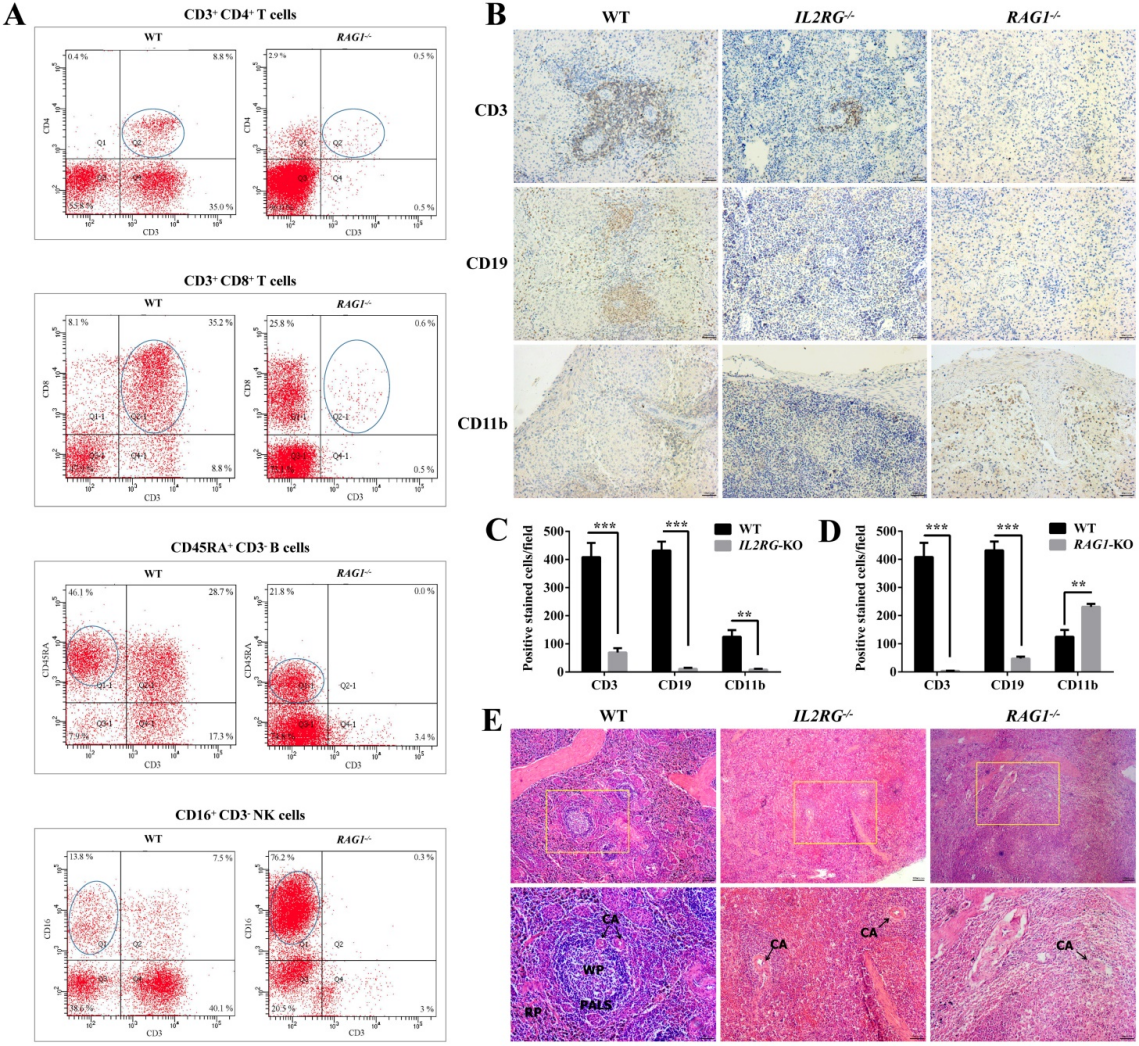
** Immunodeficient phenotype analysis of *IL2RG*-KO and *RAG1*-KO Tibet minipigs. A.** Flow cytometry analysis of the proportion of T cells, B cells, and NK cells in the peripheral blood of *RAG1*-KO Tibet minipig (piglet d13). **B**. Spleen IHC results' comparison between age-matched WT and *IL2RG*-KO (piglet c9) /*RAG1*-KO (piglet d14) Tibet minipigs. Compared to WT, CD3^+^ T cells decreased significantly in the *IL2RG*-KO pig spleen, while CD19^+^ B cells, CD11b^+^ NK cells and macrophages were barely detected. In *RAG1*-KO Tibet minipigs, CD3^+^ T cells and CD19^+^ B cells were almost all gone or decreased significantly, while CD11b^+^ NK cells and macrophages increased. Scale bars: 50 µm. **C.** Quantitative analysis of CD3-positive, CD19-positive, or CD11b-positive cells on the spleen'IHC in WT and *IL2RG-KO* Tibet minipig (piglet c9) (n=18 fields, 6 fields were taken from each section, and 3 sections were taken from each group). *p < 0.05; **p < 0.01; ***p < 0.001. **D.** Quantitative analysis of CD3-positive, CD19-positive, or CD11b-positive cells on the spleen'IHC in WT and *RAG1-KO* Tibet minipig (piglet c9) (n=18 fields, 6 fields were taken from each section, and 3 sections were taken from each group). *p < 0.05; **p < 0.01; ***p < 0.001. **E.** H&E staining results of spleen tissues in *IL2RG*-KO (piglet c9) and *RAG1*-KO (piglet d14) Tibet minipigs. Spleen tissues of age-matched WT Tibet minipigs were used as controls. In the *IL2RG*-KO Tibet minipig or *RAG1*-KO Tibet minipig, the peripheral lymphatic sheaths in the spleens were hypoplastic, and the white pulp and red pulp could not be easily identified. Scale bars of low and high magnification images are 100 µm and 50 µm, respectively. CA, central artery; PALS, periarterial lymphatic sheaths; WP, white pulp; RP, red pulp.

Furthermore, flow cytometric analysis of the lymphocyte population was performed to differentiate between WT and *RAG1*-KO Tibet minipigs (*RAG1*^-/-^) (**Figure 6A**). The percentages of CD3^+^CD4^+^ T cells and CD3^+^CD8^+^ T cells in lymphocyte samples from *RAG1-*KO piglet, d13 (4 months old), were extremely low in comparison to those of their WT counterparts, at 0.5% vs. 8.8% and 0.6% vs. 35.2%, respectively. A similar situation occurred in B cells, with percentages of 21.8% vs. 46.1% in *RAG1-*KO and WT Tibet minipigs. In contrast, NK cells constituted a higher proportion of the total lymphocyte population in *RAG1*-KO Tibet minipigs than in WT Tibet minipigs, at 76.2% vs. 13.8%, respectively (**Figure 6A**). Consistently, compared to those for WT Tibet minipigs, the spleen IHC results for *IL2RG*-KO (piglet c9) and *RAG1*-KO (piglet d14) both showed an apparent decrease or almost disappearance of CD3^+^ T cells and CD19^+^ B cells, while CD11b^+^ NK cells and macrophages were barely detected in *IL2RG*-KO but seemingly upregulated in *RAG1*-KO Tibet minipigs (**Figure 6B-D**). Furthermore, H&E staining results showed that compared with those in the spleens of WT Tibet minipigs, the periarterial lymphatic sheaths in the spleens of *IL2RG*-KO Tibet minipigs (piglet c9) and *RAG1*-KO Tibet minipigs (piglet c9) were very thin, and the red pulp and white pulp could not be easily identified (**Figure 6E**).

### Germ line transmission of targeted gene mutations

**Figure 7:**
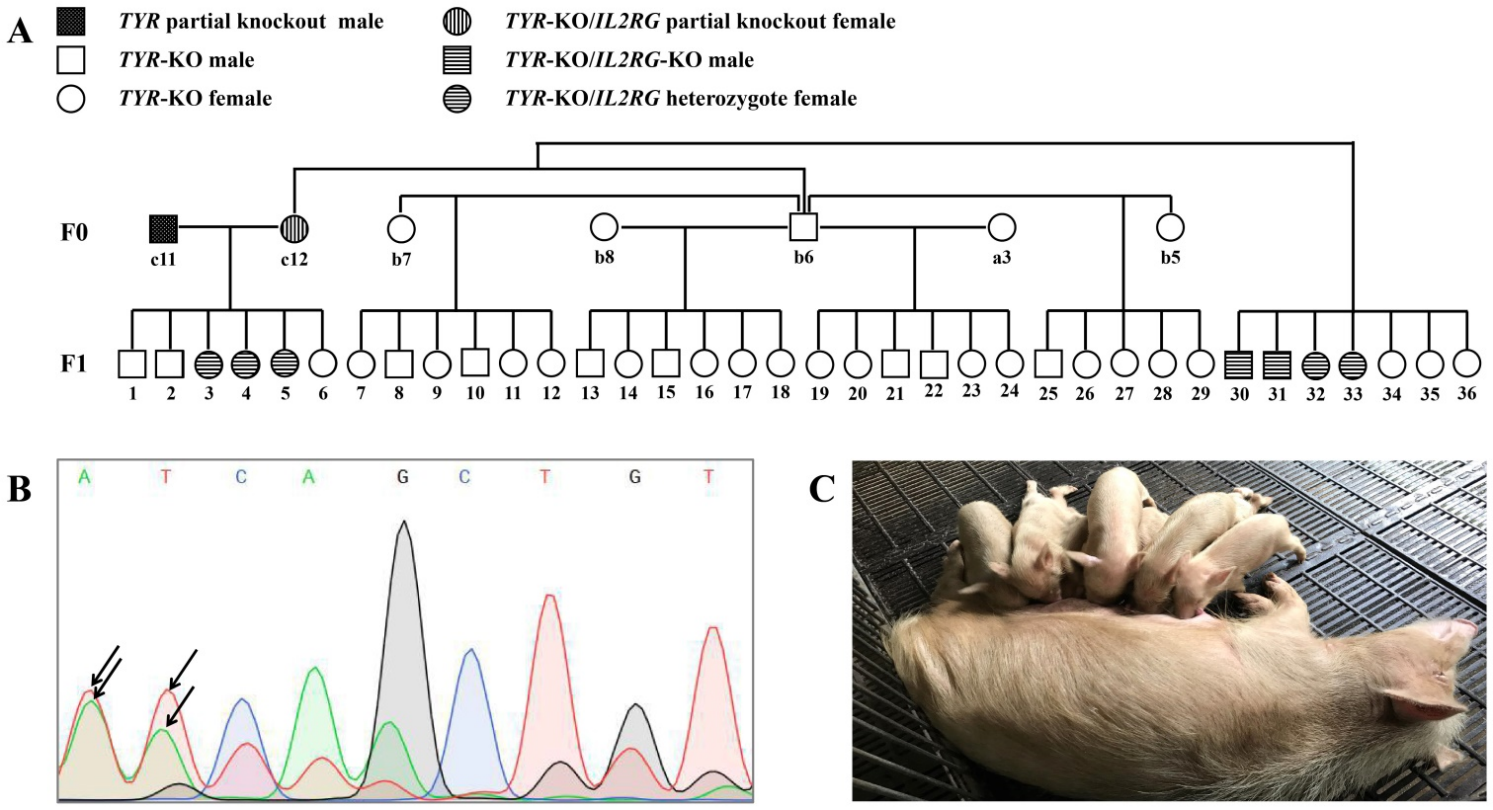
**Germ line transmission of targeted gene mutations. A.** Pedigree showing F_0_ Tibet minipigs intercross to produce F_1_ generation. **B.** Results of sequencing of PCR products showing the overlapping peaks (black arrowhead) in the targeted *TYR* locus in sperm genomic DNA sampled from piglet c11 (*TYR* chimera), demonstrating that the *TYR* mutation was present in the germ cells. **C.** F_1_ generation piglets harbouring *TYR* gene mutations are of the albino phenotype.

We next determined whether the CRISPR/Cas9-induced target gene mutations could be passed on from the F_0_ minipigs to the next generation by germ line transmission. To test this, selected F_0_ pigs were inbred, from which 36 F_1_ generation pigs were obtained (**Figure 7A**). Although the F_0_ generation piglet c11 was a *TYR* chimera, mutations of the *TYR* locus were detected in its sperm DNA by Sanger sequencing (**Figure 7B**), and all of its offspring (F_1_) piglets possessed *TYR* allelic mutations (**Table S8**). Similarly, as the F_0_ generation pig c12 was an *IL2RG* chimera (**Table S5**), it also transmitted its *IL2RG* mutation to some of its F_1_ offspring (**Table S7**). Regarding the mutant *TYR* gene's inheritance, all F_1_ generation piglets exhibited the albino phenotype (**Figure 7C**), as further confirmed by identification of *TYR* gene complete knockout by genotype data (**Table S8**).

### Off target analysis in gene-targeted Tibet minipigs

Off-target effects are a major drawback of the CRISPR/Cas9 system^28^, ^29^. Potential OTSs (**Table S3**) for sgRNAs were selected. Genomic fragments of approximately 600 bp containing the OTSs were amplified from genomic DNA extracted from skin tissue samples by PCR using the primers listed in **Table S3** and subjected to sequencing analysis. No off-target effect was detected.

## Discussion

In this study, gene-edited Tibet minipigs were successfully prepared by synchronized oestrus and cytoplasmic injection of zygotes. Synchronized oestrus treatment played an important role in the success of this study. The synchronized oestrus of Tibet minipigs can be rendered by using synthetic progestin, altrenogest, which can make the animals enter into oestrus and ovulate at the same time. This procedure provides a sufficient quantity of zygotes that can be further used to generate genetically modified Tibet minipigs by zygote injection (**Table 1**). Specifically, 5-8 zygotes can be obtained from one Tibet minipig at one time after synchronized oestrus treatment; thus, embryos obtained from 2-3 Tibetan pigs are sufficient for subsequent transplantation of gene-edited zygotes to a surrogate Tibet minipig.

In fact, we have tried other ways to overcome the limited source of zygotes, such as using different doses of Pregnant Mare Serum Gonadotropin (PMSG) and human Chorionic Gonadotropin (hCG) to induce superovulation in Tibet minipigs, only to find the results are not satisfactory (**Table 1**). As shown, the quality of embryos obtained by oestrous synchronization was superior to that obtained by superovulation. In addition, oestrous synchronization allows donors to be repeatedly used for embryo collection 2-3 times. Another advantage is that donor and surrogate pigs can reach the oestrus state simultaneously. Because of their similar endometrial environment, it is easy to transplant embryos from donors to surrogates. Additionally, after synchronized oestrus in both the surrogate sows and nanny pigs (**Figure 1B**), the nanny pigs can breastfeed some F_0_ gene-modified piglets on behalf of the surrogate sows, who may fail to fulfil their nurturing role, as experimentally observed. This approach significantly improved the survival rate of F_0_ generation pigs after birth. Practically, once we obtained enough embryos, the remaining donor pigs were kept as nanny pigs. It is worth mentioning that we adopted an additional vaginal smear test to enhance the accuracy of judgements concerning the sows' oestrus state (**Figure 2A**), by which we can select the appropriate mating time and ensure that single-cell embryos can be obtained. In summary, we optimized various steps of the whole technical procedure (see the Materials and Methods section for details), such as the sows' synchronized oestrus treatment (**Figure 1B**), a reliably controlled mating time (**Figure 2A**), timely collection of zygotes and a standardized surgical procedure (**Figure 2B-G**), all of which help make the preparation of genetically modified pigs more efficient.

Next, albino (*TYR*-KO) and/or immunodeficient (*IL2RG*-KO or *RAG1*-KO) Tibet minipigs were generated by cytoplasmic microinjection of CRISPR/Cas9 RNA. Compared to SCNT, which requires a great deal of time and effort to obtain genetically modified monoclonal cell lines as nucleus donors, cytoplasmic injection of Cas9 mRNA and sgRNA mixtures into zygotes to generate gene-edited animals is technically efficient. It takes only approximately one week to prepare Cas9 mRNA and sgRNAs. Gene editing in pig zygotes injected with CRISPR/Cas9 RNAs did not delay blastocyst development or alter the sex ratio^30^. In this study, all pregnant surrogates delivered on time, and all the piglets survived. This approach can effectively avoid the blastocyst development delay and low survival rate after birth that are typical of the SCNT method^31^. The sex ratio of the F_0_ generation was 9:7, showing no sign of gender imbalance. We also found that Tibet minipig sows were suitable and efficient as surrogate mothers of gene-edited embryos (**Table 2**).

Although the traditional one-step method (zygotic injection with Cas9 mRNA and multiple sgRNAs spaced 10-200 bp apart, targeting only a single key exon of the target gene) can efficiently generate gene knockout animals^32^, unexpected additions or deletions of large fragments of DNA or chromosome breakage often occur^33^. To overcome this problem, we designed only 1-2 sgRNAs for each target gene and still obtained satisfactory gene-editing efficiency (**Table 2**). Additionally, no large fragment DNA insertions or deletions were detected (**Tables S4-S6**). The genotype analysis (**Table S4-S6**) indicated partial population of F_0_ generation pigs are chimeras (individuals carrying more than two allelic genes): piglets a3, b8, c10, c11, d13 were identified as *TYR* chimeras, and piglet c12 is a *IL2RG* chimera. As calculated, the chimeric rate from 1-cell stage embryo microinjection was 25% while that from 2-cell stage embryo microinjection was 50%. Microinjection at 1-cell stage embryo led to a bit higher chimeric rate, and seemed more likely to generate incomplete-knockout chimeras (carrying WT allelic gene). However, we also obtained piglets (c9, d14, d15, and d16; **Table 3**) that developed from 2-cell stage embryos were not chimeras (**Table S5, Table S6**). We hypothesize that this may be attributable to the death of one cell and overgrowth by the other cell following microinjection of these 2-cell stage embryos, whereas the surviving cell eventually continued to develop into an individual animal.

*TYR-*KO Tibet minipigs had red-coloured eyes and white skin and hair (**Figure 4A-D**). Compared with wild-type Tibet minipigs, the veins underneath the semi-transparent ear skin of the *TYR-*KO Tibet minipigs were more visible, which facilitates intravenous injections. In addition, white skin is more convenient for observation of inflammation and wound healing. Laboratory animals of albino strains are not unusual, such as BALB/c mice, Wistar rats, albino guinea pigs, and New Zealand white rabbits. This study demonstrates the possibility of developing an albino* TYR-*KO Tibet minipig strain.

*IL2RG-*KO and *RAG1-*KO Tibet minipigs exhibited an apparent immunodeficiency phenotype. Postmortem examination revealed that thymus development was severely stunted in *IL2RG-*KO and *RAG1-*KO Tibet minipigs (**Figure 5E**), consistent with previous studies^18^, ^20^, ^24^. The thymus is extremely important for immune cell development^34^. In *RAG1-*KO Tibet minipigs (piglet d13, d14), the percentages of CD3^+^CD4^+^ T cells and CD3^+^CD8^+^ T cells were very low, and the number of B cells was also decreased, while that of NK cells was significantly increased (**Figure 6A, D**). This abnormal NK cell increase is likely the result of an immune compensatory response to the major loss of T and B cells. However, piglet d16, which had a 9 bp deletion in* RAG1* (**Table S6**), did not exhibit an immunodeficient phenotype. This may be attributable to the fact that the deletion was in-frame and did not cause any significant changes to the structure or function of *RAG1*. Immunodeficient pigs are extremely susceptible to death and have a short life span^18^, ^20^, ^24^, ^25^; hence, appropriate methods for breeding immunodeficient pigs are still being explored. In this study, the *IL2RG-*KO and *RAG1-*KO Tibet minipigs were maintained in a clean environment, and weaning was delayed, which allowed them to survive until 3-4 months of age. Most of the immunodeficient pigs in this study had multiple infections (**Figure 5A, B**) or unexplained weight loss (not excluding potential infection) in the later stages of their lives. We consider that the use of specific pathogen-free pigs as surrogate mothers may be preferable. The F_0_ generation piglet, d14, developed colon cancer (**Figure 5C**); since it was the single case observed, its relevance to the immunodeficiency remains to be further investigated. For the reasons discussed above, heterozygous mutant pigs are the main method used for the reproduction and preservation of immunodeficient pig lines^18^. Alternatively, the F_0_ generation piglet, c12, which was a chimera for *IL2RG* gene mutation (**Table S5**), could survive in conventional housing conditions without symptoms of immunodeficiency and transmit the *IL2RG* gene mutations to offspring (**Table S7**). Thus, we think chimeras prepared by 2-cell stage embryo injection could partially replace heterozygotes for the preservation and reproduction of immunodeficient pigs.

By intercrossing F_0_ generation pigs (**Figure 7A**), 36 F_1_ offspring were obtained. All F_1_ generation animals had the albino phenotype and, in most, *TYR* mutant alleles were parentally derived, in accordance with Mendelian inheritance; however, a few F_1_ generation animals only carried the paternal *TYR* mutant allele, with no maternal *TYR* mutant allele detected (**Table S8**). We suspect that during the embryonic development of F_1_ generation pigs, homologous recombination repair occurred between *TYR* mutant alleles, leading to duplication of the paternal mutant allele, but this requires further exploration. In fact, this new genetic phenomenon has been reported^35^. Interestingly, although minipig c11 was a *TYR* chimera, all six of its offspring were homozygous for *TYR* mutant alleles, and no *TYR* wild-type alleles were detected. This phenomenon was also reported in another similar study, suggesting that redistribution of *TYR* mutant alleles during reproduction is not necessarily proportional to the hair colour distribution of the parental chimera^15^.

In summary, our study outlined an efficient way to generate gene-edited Tibet minipigs by synchronized oestrus and zygote cytoplasmic injection. Technically, we overcame the limited source of embryos needed for gene editing and following transplantation, improved the survival rate of F_0_ generation piglets and successfully generated genetically modified Tibet minipigs with albino and/or immunodeficient phenotypes. This is the first report that gene-edited Tibet minipigs can be generated by zygote cytoplasmic injection.

## Supplementary Material

Supplementary tables.

## Figures and Tables

**Table 1:** Comparison of oestrus synchronization and superovulation in Tibet minipigs

Treatment	No. of treatment (head)	No. of oestrus (head)	Oestrus induction rate (%)	No. of operations (head)	No. of embryos	Average No. of embryos (number/head)	Embryo quality	Sows can be reused to collect embryos	Need to observe oestrus every day	Require daily medication
^1^ES	30	27	90	14	89	6.36	Good	Yes	No	Yes
^2^S	21	6	28.6	6	75	12.5	Poor	No	Yes	No
^3^ES+S	5	1	20	1	20	20	Poor	No	No	Yes

**Note: 1.** ES, oestrus synchronization. The details are described in the previous section. **2.** S, superovulation treatment. Superovulation was administered on the 14th to 15th day of the oestrus cycle. The sows' best oestrus state was set as day 0 of the oestrus cycle. The first day: intramuscular injection of PG (Prostaglandin) 500 mg at 10 a.m. and 4 p.m. respectively; the second day: intramuscular injection of PMSG (Pregnant Mare Serum Gonadotropin) 1500-750 IU at 10 a.m.; the fifth day: intramuscular injection of hCG (human Chorionic Gonadotropin) 500-750 IU at 4 p.m.; the sixth day: mating at 9 a.m. and 16 p.m.; and the seventh day: operation at 9-11 a.m. to collect embryos. **3.** ES+S, oestrus synchronization combined with superovulation. Superovulation was performed on the 14th day of oestrous synchronization. For more details, refer to Zidek V, et al^36^.

**Table 2:** Cytoplasmic microinjection of zygotes generates gene-edited pigs

Surrogate ID	Microinjected embryo cell stage	CRISPR guide pairs	Gestation (days)	No. of transferred embryos	No. of births	No. of births/ transferred embryos	Sex ratio of piglets (male: female)	*TYR-*KO	*TYR-*KO and *IL2RG-*KO	*TYR-*KO and *RAG1-*KO
A	1-cell stage	tyr-sg1/tyr-sg2	113	12	4	33.3%	2:2	3	/	/
B	1-cell stage	tyr-sg1/tyr-sg2	113	9	4	44.4%	1:3	4	/	/
C	2-cell stage	tyr-sg1/tyr-sg2/il2rg-sg1	114	11	4	36.4%	3:1	2	1 (25%)	/
D	2-cell stage	tyr-sg1/rag1-sg1/rag1-sg2	115	8	4	50%	3:1	3	/	3 (75%)
Total			114 ± 1	40	16	40%	9:7	12 (75%)	/	/

**Table 3:** F_0_ generation piglets

F_0_ generation piglet ID	Sex	Surrogate ID	Phenotypes	Genotypes
a1	Male	A	Fully pigmented	Wild-type
a2	Male	A	Albino	*TYR*-KO
a3	Female	A	Albino	*TYR*-KO
a4	Female	A	Albino	*TYR*-KO
b5	Female	B	Albino	*TYR*-KO
b6	Male	B	Albino	*TYR*-KO
b7	Female	B	Albino	*TYR*-KO
b8	Female	B	Albino	*TYR*-KO
c9	Male	C	Albino, immunodeficiency	*TYR*-KO, *IL2RG*-KO
c10	Male	C	Fully pigmented	*TYR* partial knockout, *IL2RG* partial knockout
c11	Male	C	Partial pigment loss	*TYR* partial knockout
c12	Female	C	Albino	*TYR*-KO, *IL2RG* partial knockout
d13	Male	D	Partial pigment loss, immunodeficiency	*TYR* partial knockout, *RAG1*-KO
d14	Male	D	Albino, immunodeficiency	*TYR*-KO, *RAG1*-KO
d15	Male	D	Albino, immunodeficiency	*TYR*-KO, *RAG1*-KO
d16	Female	D	Albino	*TYR*-KO, *RAG1* missing 9 bp

## Abbreviations

*TYR*: tyrosinase gene
Cas9: clustered regularly interspaced short palindromic repeat sequences associated protein 9
sgRNA: single-guide RNA
*TYR*^-/-^: *TYR* gene knockout
*IL2RG*: interleukin 2 receptor gamma gene
*RAG1*: recombinant activating gene 1
*IL2RG*^-/-^: *IL2RG* gene knockout
*RAG1*^-/-^: *RAG1* gene knockout
SCNT: somatic cell nuclear transfer
CRISPR: clustered regularly interspaced short palindromic repeat sequences
WT: wild-type
NK: natural killer
V(D)J recombination: recombination of numerous variable (V), diversity (D), and joining (J) gene segments
KO: knockout
IACUC: Institutional Animal Care and Use Committee
OTSs: off-target sites
PBMC: Peripheral blood mononuclear cell
pAb: primary antibodie
PMSG: Pregnant Mare Serum Gonadotropin
hCG: human Chorionic Gonadotropin
IHC: immunohistochemistry
PAM: Protospacer adjacent motif
DPBS: Dulbecco's phosphate-buffered saline solution
