# Supplementary material for: Optimization Strategy for Generating Gene-edited Tibet Minipigs by Synchronized Oestrus and Cytoplasmic Microinjection
Source: Int J Biol Sci. 2019 Oct 15;15(12):2719–32. doi: 10.7150/ijbs.35930 (PMC6854383; doi:10.7150/ijbs.35930)
Supplement: Supplementary file 1 — Supplementary tables. [file ijbsv15p2719s1.pdf]

Table S1. Primers for sgRNA synthesis

No.	Name	Sequence (5'-3')
1	tyr-sg1	CAGTGCTTTGGCAACTTCAT **GGG**
2	tyr-sg2	CCGTTCATCCACCCCGGTGA **AGG**
3	il2rg-sg1	CCCACAGACGCTAAAACTAC **AGG**
4	rag1-sg1	CTCAAGCCACAAGGAGTCGA **AGG**
5	rag1-sg2	ATGTCTCCTTCGACTCCTTG **TGG**
6	tyr-sg1-top-oligo	CCGGCAGTGCTTTGGCAACTTCAT
7	tyr-sg1-bottom-oligo	AAACATGAAGTTGCCAAAGCACTG
8	tyr-sg2-top-oligo	CCGGCCGTTCATCCACCCCGGTGA
9	tyr-sg2-bottom-oligo	AAACTCACCGGGGTGGATGAACGG
10	il2rg-sg1-top-oligo	CCGGCCCACAGACGCTAAAACTAC
11	il2rg-sg1-bottom-oligo	AAACGTAGTTTTAGCGTCTGTGGG
12	rag1-sg1-top-oligo	CCGGCTCAAGCCACAAGGAGTCGA
13	rag1-sg1-bottom-oligo	AAACTCGACTCCTTGTGGCTTGAG
14	rag1-sg2-top-oligo	CCGGATGTCTCCTTCGACTCCTTG
15	rag1-sg2-bottom-oligo	AAACCAAGGAGTCGAAGGAGACAT
16	T7-tyr-sg1-F	TTAATACGACTCACTATAGCAGTGCTTTGGCAACTTCAT
17	T7-tyr-sg2-F	TTAATACGACTCACTATAGCCGTTCATCCACCCCGGTGA
18	T7-il2rg-sg1-F	TTAATACGACTCACTATAGCCCACAGACGCTAAAACTAC
19	T7-rag1-sg1-F	TTAATACGACTCACTATAGCTCAAGCCACAAGGAGTCGA
20	T7-rag1-sg2-F	TTAATACGACTCACTATAGATGTCTCCTTCGACTCCTTG
21	sgRNA-R	AAAAGCACCGACTCGGTGCC

Note: 1-5: sgRNAs targeting the *TYR*, *IL2RG* and *RAG1* genes. Roughening marker for the PAM locus; 6-15: Oligos for the generation of sgRNA expression plasmids; 16-21: Primers for generating DNA templates of sgRNAs by PCR amplification of sgRNA plasmids.

Table S2. The primers for the target gene loci

Target gene locus	Primer	Sequence (5'-3')
TYR-exon1	TYR-TA-clone-Forward	GTACTGCCTGCTCTGGACTTT
	TYR-TA-clone-Reverse	GGATGCTGGGCTGAGTAGATT
IL2RG-exon3	IL2RG-TA-clone-Forward	TGATAAAGTCCAGGAGTGTGGC
	IL2RG-TA-clone-Reverse	ATTCACTCAGGGTGCGAAGG
RAG1-exon2	RAG1-TA-clone-Forward	TTGTGAACACATCCTGGCCG
	RAG1-TA-clone-Reverse	GATGGCCAAGCAAACAGCAG

Table S3. The primers used to amplify the potential off-target sites

No.	Potential off-target sites	Sequence (5'-3')
1	mm4_intergenic_ENSSSCG00000018662|ENSSSCG00000004870_1_165414546	TCTGACTGCTTTCCCTGTGTCTAGTCGTGAATATTGTATAAAGAGTTAAACGGTTGAGTAGTACTTACCTAATCAGGAGAGAATAAGGGCATTTACTAAATCTGTCACCTCTGCTTTCATGAAAAGATTCTAGACATTTTCTATTGTGTATTAATGTGCTTATGAACTGTTTATTCTTAGTTGGTTTAAGAAAACCTTAAATAGAAAGTTACTAATGCAGTAAAACNNNNNNNNNNNNNNNNNNNNNNNNNNNNNNNNNNNNNNNNNNGCATTACCTAGTTTTGTGCATTAGAAGGGATCTCTATGTAGATGATTATAATGTCACTTTCTGACCTTTGCAGCTTGAATAATTGTAAATCATTGCTTCATAACGTGTCAGAGAATGTCTGTACTAGGCAATTAAATGGCAGAATATAATAGACCAAATTATTGCCTGTCATGTTTTCAACAACACAGTTAGTATTTGAGTTACACTTAATCATT**ACGTGCATTAGCAACTTCATTGG**AATCCAGCTTCAGGCTGAGCATGAAA
2	mm4_intergenic_U6|TUSC1_1_219020557	TGAGGGTGACGTGCACTTTTAAAGAAATATTCTGCTAACTACATTAGCTCTCTATGTAATGAAGAATGCATGCATAGAAGCCCATAATGACTACCTGGCAGTCCCCTCTGCAATTCATTGAGTTTATAAATAAATTTATTCCTCTTCTGTCTGGTTAAAAAACAAATATTCCCTCATTTTTTTCAATACACAGCATATTAACTCCATGGTTCAAAAATTATGGATTTAGTTTCTATCTTTAAACTCTGCTGTATTATGTTTATAATATAGATAAATATCAAAAGTATTCAAATATCGGTAAATTAGAGCCAGGAAGTAACTTGTTTAGT**CCAATGATGTTTCCAAAGCACCA**GAGTCAAGAAATCTGAGATTATAGCACTAGCATTGCAAAACATAAGTACCACATGAAGTGCAATTTTTTAAAACTGNNNNNNNNNNNNNNNNNNACTGCTGTTATTCCTGTAAGGTAGGCTACTATTAGAGTCAGCTTGTCTAGTCTTCTGAATACATGGAGGGCAGTGGAGA
3	mm4_intron_PRKDC_4_87291457	AGACTACACTGCAGTCACGCATGGAGATAAGACTCGGTTCTCATAAATTCGAGCTTTGTGAGTTACAGGGCATGTGTC**CAGTGCTGATGAAACTTCATAGG**AGGCTCAGACCTCATACAGTCTGTCAGCTCACCTGCAGCTGTGCTGCAGTTGTTAATTCACTGAGCTTTTAGAAATTGTATTTGGACTTCATAAATAATCGCAAATTGTAAAGCATAATCTGCTTTTTAACTAGATTAGTTTGAATATTTTATATTCATATTATGTTTTGAATACTTTATGGCACTGATTTTTGGAGTCAGTTTTCTTAACAAGGCAGTGAAGGACTGTATATCNNNNNNNNCCACATTTTCTCAGTTTGAAAACAGCTAGTGGTATATACTTATCATCGAAAACTTCCCATGTAGTAGAGATGAAAATAACATCTCCCAGTTTCTCCCCTCTTGTTAAACGTGCTAGCATTTCTCCCCTTGAAGGGATGCGTTGTTTGTAAGTCAGCTCGCTGTGTTGGTAATAGTTTTGCGTGTGGGTTCCCTGCCTTGAGCCCCTGGTGGAGGAAGAGGTGCCTTC
4	mm4_intron_CCNI2_2_140545251	TCTGAGGCCTCTGTCTCTCCCTCCCTCCAACTTCTCCTCCTTTGGGGATATGGAACCCCTGCTATTTCTACAAGGTCTCACCCTGAGATTGGCCAATCTCAGGGTCTAAGCAGGCAGAAAGAGTCTCCTGTGTGTCCAGTTCCAAATCTTTGGGNNNNNNNNTTTAAATCCTGTTTGTTAGAATATAATGTAAGCCTCCCCCCAAGATGTTTTGTCCAACTAAAAATCAAGTAAATAACCTTTATAAAAGAACTGTTCCTAGAGGTTGTAGTAAGATTTTGTTAATTGNNNNNNNNNNNNGTAGGACGAAGTCTTTGATGCACTTGGTACTTGAATGTTTATTCACAGACAAACTTTTGTAGTCTATGTGGAAAAAAGTCAGTTTTTCTGGGACTGACCTGCCTTCAATAAAGGCAGAAAGGAATGTGTTTGTA**CCTATGGTGTTGCCAAAACCCTG**CGAGGTTTTATCAGCGTCTCTTTCTCATAGAGAGAAAGGCATACCCTCTATGACAAGGAATTACGGTAGGTCTGGAGATGGAGAATGGGTTGAGGCCACAGCTGGAAAGCTTTGAGTGCGTTCTTGCA
5	mm3_intron_MYH15_13_160151996	CCTTGCAGGAGATGTGAGCAAGAAAATACATCACA**CCCATTAAGATGCCTAAGCACTG**CGTTGTGGGGCCCATGGAGGAAGCATTTTAATCAGCCCCTCCATCTCAAAGGATGCTGAGGAGGATGACAGACAGCGGCTAAAGTGAACCCAAGAACAGTGGAACCTATAAAGTGAGCATGACACTGGGATGGATTTGAATATATAAATTTGGAAGAGGTGCTGAAATAGAAAAGAAATTCAGAGAAAAGAAGATTTGGGCATATTTAGGACTCAAATTGTGAGCAAGGGAGAGAGTGAGCGGGAGAGAGCCAAAGAATCTTTCCTGGAAGCAATGTAGCCAAGGCGCTCTTTAGAGACTAGACAAGCTAAAGACTTTTAACAGTTGCTTACTATTGGATATTCACTACTATATTCTTCTTGCTCCCATTTCTTATGAAGTCCTCAATATAGTCAGGGGAAGCAGGGATTCAGAGATCCTGAGTTTCAAGTCCAGGTTGGCAAAGAGTAGGGGAAAATAAAGTACATGTGGACACAGGAGCCAGAGGGTGGTGGTCAGAGACAT
6	mm4_intron_RALGPS1_1_301465549	AAGTGCATGGACTTTGGGCTCGGTTCTGTGACCTGGGCA**CCCATGATGCTGCCAAGGGACTG**AGCTGGTGAGGGGCGCGAAGCTCCCCCCAGCCCTCTTGGCACTCGTGCCCCTCCACGAGGGACCCGTAGCTCCTGCACATTTAGTCTGACTCCCCAAGTGAGGACGTTAAAGACGACCTGACCCCAACCCAGAGCCTGAATTCCAGCTTAGCTTGACTCCACCCAGGTGACCCCAGCAGAGATCGGCTGAACTGCAGTCCCTCGACTGACCTATGAACTAAATGTTGTTGCTGTGAGCCTGAGGTTTCAGGGATGTTTGTGATAGCACATGATCAGCTGGAAACACGACCAAAGCAGTGTTGATCTTTCCTGTTCAGATCTGGCATCCAGGATTGTTAACTTAACTCCCTTGGTTTTTGCGTGTTCTCTTTTACACTGACACTTTTGGTTCCTGGCAACAATGAATAATTACCTTTTTGTATTATCTCATCATATGTATAAAGGAGTTTTAAAATGATAACATCAGTAGTACAGTGAACCAGCAGGTGAC
7	mm4_intergenic_ENSSSCG00000001905|ENSSSCG00000001909_7_63882149	ACTGGATGGATGGTGCCTTGGCCCTAGAGTGGGAGGGATATGGTGGGTGGGAGGTGGGGAGGCCGGTTTGGAGGAGCCAAGGACCCCTCTCCTCTGTATGCTCACTCTCTCCCGCAACCCTGCTCTTGTGAAGGGTCTGAGCTGCCTCTTTC**CAGGGCTATGGCAGCTTCTTGGG**ACTTCGGTTCCACTGAGGAGGTTTGAAGAGCCTCATGGGGAGGGCAGGTATATAGCCTCCACACAACTTCCAACCCTTCCCCCCATCTCCCCGCCCATATCCACCCGGGCCCCACTCACTGAAACTCCTTGTAGTTGGGAACCACCCGGGCAACGACCACCATGGGGCTGGGGTTATTGGCCAGGGGCTCATTGATGCCCCAAAGAAAGACCTGGGGGAGAGAGGAGACAGGTGAGCCTGGGGTGGCCTTGGGCCCTGTCAGGGACATGGCCAGCCCACCATCTTTCTCCCCAACAATGAGATCATCCCTCTATCCACACCTCTGCTCCTGTCTCACCAGGTTCTTCAACAGGGGCTCAAGGCTGCCCTCTTTCA
8	mm4_intergenic_KAL1|CH242-37L1.1_X_5607463	AGCTATACCCCACGAGCTGAAAGAGCAGGTAGTGGAAAGACTGAGAGGGTGGAAGGATGGGGCTGGGCAATGGTCACCTCTGGTTCCTCTGAGTCACCTTCTCATCAGGGCCTCAGCCACAACCTCACGTTCACTCCTTCAAAACGCAAGTTTGCTCTCAAGGAATGCAACTTGAAAACTGAGGCGCACCGAAGGCTTGGGAGGTCAAGGACCCTGTTATTTCAAGGTCAACTCGCTGTTAACCAAATGAATGGACTTAGATGTGACATT**CCCATCAAGTTGCTAAAGCAGAG**CCCCAAGCAAAACATTTCAGAGCAGGGTTTATAGTCTGTAAGAGGATTTTTACGAGTGGGACGTTACAGCTGCTACCATACGTGAATGGACAAATACATACGCTCAAAAACAGCATTCGCTAACATCAGGCGGAAACCCACAGTCTCAGTACATCAGGTTCTGGGGACAGGTGAGAGGTCAGGTGAGACAGAAGCCCAG
9	mm4_exon_CH242-196P17.1_1_64803335	TCACTGTTGTCAGGCACCTCTGAGACCAGTAAAGACTAATGACATAAATAAGTCATTAACATTCTGCTTTTTGAAAAAATCCTATAAAAGTAATAACTTACCTCAATATTTACTTTGGGGGTTATATTTAGTATGAAAATGTTAAATGACAAAGACTCTAGTTGTCAAGTGACTATAATATACTTTTCTCTTCCACAGATGTGTTTCCACCAAATTCAGCTCATTCTACCTCCAAGACTCAATCGGATCTCAACAAGGAAAATAAAAAACCAACTGACAAATCTGACAAGTTTACCGAAGCAGACTCCAACAAGAACTCATCTTTAGATGAACTAGAAGAAGGAGAAATTATAAGTGACAGCGAAAAACCTAAGCCACAAAAGAGTTTTGAGAAAAGTGCCAACCCAAGAGCTTCTGCTCAAATGCCGAGCACAAAAGCTAA**CCCAGGAAGTAGGAAAAGCACTG**TGCATTTGGATAAAGACAATAGGAAGATATCTTCTATAAAAATCCATCAGACCAAAAGCAAATGGAATAAGAGACGCAGTGAATCAAGCAGAGC
10	mm4_intergenic_ARRB1|ssc-mir-326_9_10765474	CCAACTCCAGCCTGATTCGTCACCCCCCTTCTCCTCCCCTCACTGCCACTTACCAACGTTAGGGC**CCGGGGAAGTTGCCAAACCTCTG**ATCCCTCCCAGACATCACTGGATCCCATGACCCCTGGTTTGCTCCTGGAGAGATGCCATCCCATCTTCTCTTTGGCACTACCGACCACTTCAGCTGGAATCCTGCCCCAGTCTCAGCCCTGGTTCTTTTTCAGTTCTCATCTGCTGCTCGGCTGGGTCTCCCTAAAGTCACTTCAGTTTCCGTGACTTACCTTTACCTGTCCCAAAAGAACACATGGGAGGAGTAGGGATATAGGCAAGGAAGATTCTGAGTCCTACATGACAACGTATTGTATTAATACAAAAGGAAGATGCTGCTGAACATCAGATGACATTTCTAAAAAATTGGCCATATATATGAAGCAANNNNNNNNNNNNACATTCCT

		GTAAAATAATGTGCAACTTAGATCAGTGCCTCGTCCTCT
11	mm4_intergenic_GRXCR1|YIPF7_8_35561499	ACCTGGACCTGGAGTCTCTCTTTTCAGTGAGCAGCATTCAGCTTCAATGATGACAAGAAAGAAAGCATTAGACCC<br>CTTATTTCTGAATAATTTGAGTCCAAAAATACTCTAGATAGTTCTTTCTCTTCCTTGCTGTTATTTCTCAGAATCAG<br>CCAACCTTACTTTTTTGAACACTTTAATGATAGAACAATAAATAGGCAACTTACCCACCTCGGAGCAAGAGGTTC<br>TTCCATCTCCAGGCCTGTAGAGATAAGTGCATTTCCTCAGTCATTTTACCCCTTCTTGCTGCCATCTGCAGCCTCA<br>GGACACCATGGTT**CCTTCACTGGGGTGGGTGGACAG**GAGCCTCTGCACCACTTCTGTTTAAAGCTCAAAGTTAT<br>CGTCTTATAGGGGTTGTGGTTTATCACCAGAACCCAAACAAAGAGAATTGTCTTTCAAATCTCCAAAGTTGTGCC<br>TGAGCAACACTCCTTGTGCCTGGAGATTTACAAAGCCTTTTATAAAGACAGCTTTTTGGCTTATCCCTTCTGACCC<br>TTCTTGTCCACAAATACTAGCATTTTACTGCCAACTCCATCCTTCTCCATCCCGCCTTTCCAAGAA
12	mm4_intergenic_7SK|CXCL11_8_75897346	TTCTTGGAAAGGCGGGATGGAGAAGGATGGAGTTGGCAGTAAAATGCTAGTATTTGTGGACAAGAAGGGTCAGA<br>AGGGATAAGCCAAAAAGCTGTCTTTATAAAAGGCTTTGTAAATCTCCAGGCACAAGGAGTGTTGCTCAGGCACA<br>ACTTTGGAGATTTGAAAGACAATTCTCTTTGTTTGGGTTCTGGTGATAAACCACAACCCCTATAAGACGATAACTT<br>TGAGCTTTAAACAGAAGTGGTGCAGAGGCTC**CTGTCCACCCACCCCAGTGAAGG**AACCATGGTGTCCTGAGGC<br>TGCAGATGGCAGCAAGAAGGGGTAAAATGACTGAGGAAATGCACTTATCTCTACAGGCCTGGAGATGGAAGAAC<br>CTCTTGCTCCGAGGTGGGTAAGTTGCCTATTTATTGTTCTATCATTAAAGTGTTCAAAAAAGTAAGGTTGGCTGAT<br>TCTGAGAAATAACAGCAAGGAAGAGAAAGAACTATCTAGAGTATTTTTGGACTCAAATTATTCAGAAATAAGGG<br>GTCTAATGCTTTCTTTCTTGTCATCATTGAAGCTGAATGCTGCTCACTGAAAAGAGAGACTCCAGGTCCAGGT
13	mm3_intergenic_ENSSSCG00000015055|REXO2_9_46805059	CACCTTGTCGTAGCCTTGGTTCCTTGCCCTCCAGAGAGCTGACTCC**CCTTCACTGGGGTGGATGAGCAG**GAGAG<br>ACAGAGACACCTGGCCCTCCCGGAACAGAGTGAAGCTGACAAGGTAGGTGCCATTGTTGAGGTCGCTCACCTTTC<br>CCGAGGCGCCTGCCTGCAGGGCTGGGGAGGACATCCTGGCCCTCAGGAAATCCCCGCCATATGGCTTCTGCATCC<br>CAAGTGGTCCCTCACCTCCAGCAGGACATCCGGCTGATCCCCCCTGCAGTATGTGTCCTTAGGGCTGAGGATGGT<br>GGCTGTGCTGTGTGTGGCGCTGGTGGTGGAGGTCACGTGGGTGAAGGGTCTGGGTGGGAACAGTTGATCGTGATT<br>CCTTTTATTCTTAGCTTGGTCTCTGTTGGTGAATCTGCCGGATCCAGTGGTGCTATATGGAGTAAGACCACTTTGA<br>AGGTATTTCTGTGATGCATAAAGGTAGGCACCTCTTAGTCACACTAAACTTAATGTGACATATGTACACACCTGA<br>GCCGGTCTCTTTT
14	mm4_exon_ENSSSCG00000026203_9_47067492	ACTACTGGAGAGTGGGCACTGTCCCCAGATGAACGTATAGATCTTTTCTAAAATGGCTCTTCATAGAGTCCCAAA<br>GTTGATATACTCATGCTCGTTTGGCTAAATCAAAGGATCAACATTTGGCTAAATCATTACTGTATTTGCTCAGGTA<br>GTCTCCAGAAGTAATTACTTACCTTTCAAAGAGGCTCCTTTCTTCTTTGCTAAAATAAGACGCTTTCTTGTGTTTGG<br>AATCCATGTGGGTTAAGTGCAGCACAGGGTATGTCCTGAGGCTTCAGGCAGTAGAAGGCTCCCTGGTCTCTGGTA<br>TCCAGGTACTCACACAGTTAACCTCTTGAATTTAAAACCAGAGCACAAATACTGTTGACTTGGGAGGTACCACTG<br>GCAAAGTGGCCTGTGAAGATCACCTTGTTGTAGCCTTGGTTCCTTGCCCTCCAGAGAGCTGACACC**CCTTCACTG**<br>**GGCTGGATGAGCAG**GAGAGACAGAGACACCTGGCCCTCCCGGAACAGAGTGAAGCTGACAAGGTAGGTGCCAT
15	mm4_intergenic_MYC|FAM84B_4_12886286	CTCCTAGCCCAATGAATCTGCTTGAAT**CCATCACCTGGGTGGATTCACAG**TGATGCACCAGCCCTCTCATGTCT<br>CCCCATTGCTACTTTCAGACTTTTTTGCTCCTTCCTCCTACCATGCTGCCTTCTCCTTGGAGGCCTAGGCCATCTTT<br>CAACTGTTTCTATTGTCCCTTTCCCACCATCCCTGGAGACGTGGGTCCTGGCTCAGGGTCTTCCTTTCACCTCACTT<br>TTTCCATCATCACGACTGACTGCAGTGCCCATAAGAACAATGCATTCAGCCTCCCTGCTTCTTCATTCCTTGACCT<br>TGTCAACTCCCTGTGGTCTTCACCTCCGCTCCAGCCCAACTCCAAGGTCATACTCTGTCTAATGTCAGCACCAAAT<br>GCTCCATCTCAGCGACCTTCACCTTCAGCTTCTCCTGCTCTGATGCTGGCTTCTCAAAGTCTGGCCCCTTCATGTTT<br>CCCCTCTATTTCCTCTTGGCCCTTCAGTGTCTCCCACCTTCACTGGTTCGTGACTCAGCCTAGGTCCCCTGCTTGA<br>TTATCGGAAGCCTTGACTTCCTCAGCCTCTGCCACAACCATTCAGCAAGGCCCCGAATCTTGTCAG
16	mm4_intergenic_Gap|POR_GL894019.2_2673	TCTCAGTGACCTCTCCTGGGGCCCTACCCACCCATTCCTTTTGCCTGAGCTCAGCCTTTTATGTCACCTCTTATTTG<br>ACGCTTAGGTAAGATCACTGCTGTCTGGAGTTACTTTGATCCACGAGACATGCC**CCGTCACCGTGCTGGGTGAA**<br>**CTG**GGTCTGTACAAAGGTTTGAGGACCAGCTAAGAACACATGAACTTATGTGCACCTTCTCTGAACCCAGTTGTG<br>CTGACTCTGTAGGTCACTTGGGGTTGGTTTTCAACGCAAACAAGGGTGGCGGTTCCTGACAACGCAGGAAAATGG<br>ACCAACCAATTGTGGTGGCTTCTCTCGGAGGTGGGGTTTAGGGTGGGACACGACGTTCCAGGCCTTAGAATGTGT<br>GGTTTTGTGAAGAAGGCATCTGTTGGTTTCAGTTAAGGGTGTCAGAACCTGTGCGATTAAGAAACCACCTGGCAT<br>TACCTGGACATCGTGGACCCAGTCCCCTATGCAATAGGATTTGCCAACTCTAAAAGGTCACTCACCACGACC
17	mm3_intergenic_ZNF251|C8orf33_4_75151	GGGGACATGGGAGGAAAAGGCCAGGCTAGAGGGGAGTGGGTGCCCAGGTGGAGCCATGGGGTTGGGGTCTTTCT<br>GGGTCTGAGCTGGGAATGGACGAGCAGGTGGTGAGTGTGACCCAGGAAAGCTGCCCTGGTGACACAAGTCTGAA<br>CCCACTTCACAGGGAAAGGCCTCCTGGGTGACCCCAAAACAGGATTGTAAGACAAATCCCAGCAAAGGAAGGAT<br>TTGTAAAACAAACCCCAGTGAGGGGAAGGATTGCACTGCCCCTAAGAGGAGGCTCTGAGAGCTGGTCCCCTCAT<br>GGGGGCACGTGGAACCTGCCGGGAGCGGG**CCGTCACCTGGGTGGATGGAGGG**TGCAGACCCCACTCCGTGGC<br>CCCACATCTGCGCTTCGGTGGGTTACTTGGCTTCTCCGGGCCTCGGCTTCCCAGCCTGCTGGTGGGGTGACAGAA<br>GCACGGGCTGACTAGTGATTTGCTCCTAACGGGGCGGCAAGGGGTCTTGGGAAATCTCCGGGAACG
18	mm4_intron_HS6ST2_X_124987558	ATTGCACTTTCCCCTCCAGGTTTTCCATTAGCTGCAGCCATTAGAAAGAGCCTGAACCTTGTCTTTATGAGGCCTC<br>CTTCAAAGTGGG**CTGTCACCGGGGTTGATGGTCCG**GTTTACAAGATTGAAGACTTGGGCTCTGTAAAGATCTG<br>CTCTTGAAAAGTCATTAGGGAGGGCCCCGGGCCCAAGCTTTTACACCTGATGCTGAAATTCCCGGGGCATTTTAC<br>CCCCGTGAACACACACATTCCACTCACCCACAAGTAATTTGCTTCCGCTGGGCATGGGAGTAGTTACCTATGCAA<br>AATACAACAGGAGGTAGCGGGATGCAGGCGGGGACGGGATGCTGGGGGTTGTTTTGTGTATTTTTCAGACTTCAA<br>AGAAGAACAATTCTCAAGAAACTGTTTCAGGACTTTGCATTTTTGCAGAATTTTTCTTCTTTTGCGATCTCTGAGA<br>CCGAACGATCAATCCTGTGACCGGTCTATACCCGGGGACCTGTCAATACCCTGCACG
19	mm4_intergenic_KCNIP1|KCNMB1_16_57833329	AGAGCAATTCAAGGCCGTCAATTGCTAACTCACGTAACTCACGCAGGACAAACGGTGAGGGCCTGGGTGGGAGC<br>GGGGAGCGGATGTGATGTTGGCGCTTATCGCTGGGATGTGGGGGAGGTGGTCTCTGGGAGACTTACGGGGAGGA<br>GACCGGGCAGGGGGAGAGGGGACAGTCCCGATCTGCACTTACTTTGTCCTTTGCTCATCCTTGCTCTCTACTGCCT<br>TCCAGGAG**CCGCTCCTCCACCTCGGGGAAGG**TGCACAGCCTGAGCCAGGTCGTCGGGTCCATTCTCTTCCCTTC<br>CGTTTCCGATGACAACTTTTCTGGTGACCTCCTCCAAGCCACAACCACTCGAAACATCACGGTTGTTTTCCATTCC<br>TTCTCCTTTCTTGTTACCGACTTCACTGGCACTGCTTGCGCTCTTCCTTCGGCCAGCAACTTCACCCCCTGCCTTCC<br>TGCCTGGGCCAAGCCCTCGCTCTCTGATCGGCCTCAAGCACCCCCTGTTCCAGCCTTCCTCTCTGACTCTCCAGCC<br>CT
20	mm4_intergenic_SNORA51|U6_13_200586772	TGAGACATCTTTCCTGCTGTGTTAGGAAATGATGATTTTTAGAATTCTACTGCCCATTTTAGTTTATAAATTATCCA<br>TTTAAAAATGAGTTA**CAGTTCATCCCCCCCGGGGTTGG**TTACTAGGGTATAGGATCACTGATATTATTATTGTC<br>TTGTTACCAAATAAGGACAAGGGTCATTTTTATCCACTTCTTTTACCCATTAAATCAGGGTATGATTAAATAAAAT<br>TCCATGAATAACTTGATCACTATGAAATGAAATCTGCCTCACTATGCTTGCATTCAATTGGTTACATCAGCTGCTG<br>TTTCAGTGCTCTTTTCTTAACAACAATAAAATACTCAGCCTTGATATTCCCATAGAGAATAGAACTAATTCTTTTG<br>ACCTAGAGCTATAACTAACGGCAAAAGAGTAGAAAAATCACACCCATGTTAAATAGATACATTATTCTTACCTCT<br>AAAAAGACAAATACTGGATGCATTATTAAAAATGAAGTCATGTTTTAATATTTTTCAGCTCAAGAACTAAGTGCT<br>AATTTATTTCTTCCCTCAATTTGTTGAAAAGTATTTTTATTGCCTGAGGCACTACTCAGGAAATCAGGA
21	mm4_intergenic_ENSSSCG00000025926|ENSSSCG00000011605_13_7792426	GGTTTTGCAGCCTTCACCTGTGGCCATAGAGGGTATTTTTATATCTTATTTAACATGAGTGAGAGCTTGAACCTGT<br>GAGCCTGGGAGTCATGGCCCATAAAACCAGTGTTACCTGGGAACCATGTGTTCTGTCTGAGAACTGACCCAGGGG<br>TGAGGGGCCGTCACACCTGATGGAGAGAGGACAGGCAGAGCCACCGTGAGCTCAAAGGAGGTGCCAGGAGGCT<br>CTTGCCTAGACAGTGGCATTGCTGAGTCTGAAATGCAATTGTACTAAGTTGATCCTCAGCTCAAGTGAAAAAACA<br>ATATTTTCTACCATAACGTGGCCCCTCTGATAACCACAGGGAACTGACAGACACCTTTGGAGCACAGCTCTTCCC<br>AGATCATTGTTTTCCAAGAGAGAATAAAGTTAAGAGACAAGTAAAATGTTCTTCTGGGGACGCCGTGGTTCTGTC<br>TCCCCAGCTGGGATCTTATCACTTT**TCTACAGACACTAAAATTACAGG**CCTGCAGAGAGGTAAGCTCATGACCT

	0	CCTTGCC
22	mm4_intron_LEO1_1_132911974	AGTTCTGCCTGAGAGGAGGGGGATCTGGATAGACATGGA**CCTGTAATTTTAGCACCTGAGGG**ACTATGTAGAGAATATGCCTGTGTGGAGTTCTTAAGCCCCAGATTTGCATATATACACTGTAGGAGGATATTTAGGCTCCTGCGTAATAAAAGGAAGTGGAAGAACAAGGCTTTGTAGTAAGTGAAGGGAGAAAGGAGTATGTCCTGTGCAGCTTTGCCCTTCGCTGGATCTGCCCACTTCCCTCTCCACAGACACTCAAGTCAGATCTTTGAGAGCCAGGCTAAGAGCTTTGTTTGCGTGGTTTCCAGGTATCTGGTTGAGAAGCCTTGGGAACATGTTGAAGTGATAGCTCTGGAAAATCATTATTGTAGATATTAATACTCTGCACTTTTCCTCTGTTGAAGATAATAATGGAACTATGGATCTGTTTGGAGGTGCAGATGATATATCTTCAGGGAGTGATGGAGAAGATAAACCACCTACTCCAGGACAACCTGTTGTAAGTAAAATTAGCAATGTGAGCTGGGGAGAGAACT
23	mm4_intron_ENSSSCG00000023000_11_77173365	TCTGTCCAGCAGCTGTCAAGGTTGAATTCACTGACGCAAGCTGGAGCTACCCATTGAGAGGAAATTACTAAAATTACCTCCTCACTGCTTCTACCCTGATTCTTCTGAACTGTTATCGTCTGCTGAATTGAAATCCTATGCTAACCCCC**CCAACCTACGCTAAAACAACTGG**CAACGTAATTTTCCTAAATCCCTTCTGTAATTCCTGACATAATTCTTCCTTCCACCAGAATAACCCATAATGACAAGTACGTTAGTTAGACAGTTTTTTTGGTATAGTATTTTTCCATGAAATTTTATCATTTTGGTATTAATACCGTTTCTCAGAGAATGGGAGTGTTAGCTTCCAGAAGTTCACAAATTATATTTTAACTATGTTAAAAACAAGGCATTTGCTCTCAGGAGAATTGGAATCAGGAATTCCAGTAGGAAAATTGGTGGGATTATGGGTATTTATACATAATCACATAATAGTTTGCCAAATTGTTTTTAGTGCCTTTTAGTGTCTTTGAAAAGTATCTTGTTTTCCCACCAGGCA
24	mm4_intergenic_ENSSSCG00000020902|ENSSSCG00000025382_GL893949.2_212771	TGCCTGGTGGGAAAACAAGATACTTTTCAAAGACACTAAAATTCACTAAAAACAATTTGGCAAACTATTATGTGATTATGTATAAATACCCATAATCCCACCAATTTTCCTACTGGAATTCCTGATTCCAATTCTCCTGAGAGCAAATGCCTTGTTTTTAACATAGTTAAAATATAATTTGTGAACTTCTGGAAGCTAACACTCCCATTCTCTGAGAAACGGTATTAATACCAAAATGATAAAATTTCATGGAAAAATACTATACCAAAAAAACTGTCTAACTAACGTACTTGTCATTATGGGTTATTCTGGTGGAAGGAAGAATTATGTCAGGAATTACAGAAGGGATTTAGGAAAATTACGTTG**CCAGTTGTTTTAGCGTAGGTTGG**GGGGGTTAGCATAGGATTTCAATTCAGCAGACGATAACAGTTCAGAAGAATCAGGGTAGAAGCAGTGAGGAGGTAATTTTAGTAATTTCCTCTCAATGGGTAGCTCCAGCTTGCGTCAGTGAATTCAACCTTGACAGCTGCTGGACAGA
25	mm4_intergenic_CH242-112D2.1|U6_X_35039502	AGGTTTCCTTTCCTGCCAAATTTAAATTATAATTGTTTTTATCCTTCTAAATTCCATTTTGTTTTTGACTTGGCAAATATGCCATAAAAAGAAAAATGCCTCTA**CCTGTAGCTTTAGTGTCTATGGT**TTCACTAGCATTGGACTTTGAAACTAGTCACATTCCAGAACATTCTATCAAGAAAAAAAGGTCAAAATCCGTGAGACAGAATTCTTACATTGCAGTCCAGATTTTGAAAGAAGACTCAATTTTGCTGTTCATTAAACTGAGTCATTTTTTTGTTACTTGTACTTACAGATACCTACAGCATTGCTCTGAAGTTTGATTCTAGTTAATATGCGATATCATAAATTATTCAAAATAAAGAGTAAAAAAATCTTTGAGACAACTCAGGTGAAATCTACATGACAAAGCAACTGTTTAATCAAGAAGATGCAAGGGACTAAAATGTTAGACTTCTAGAGGGACCATTTACTAGTATTTGGCAATATTAATGTTTGATATCTATAAATAAGTTAACAGAAAATACCTAAAATCTCCTATTATGGTATGGTATGATATTATTGCGGCCCAAGG
26	mm4_intergenic_U6|ENSSSCG00000029792_16_3113621	CTACCCACCCTGCTTTCCAGATCAGGAGCTACACCTAATTGTGCTCAATGCTACCAAGTACTGACTTGCAAATATAAAACCCCCACCTCTGTTCAAGGCTCATGATTCTCTAGGTGCTTCAAATAATTTTTTAATGGAATAAATATTTTTAAGTTGCTGCATAACATTTTTCATCAAATAAAGATGAACTTTCAAATGATGGTCTAAATTTTAAAAATTGCCACATTTTAGAAAGGTTTACGGTTCCTTTTTTATTTCCTTGATCCCTAGAATCCATGATTAATTACATGCAAGTTACTTAGAGCCTGGAGATTTTCATCTTTCATACAGTTTCACAGCATCAACAAAAAAGTTGCAACTCATGGGCACAGACTGCCAAGAAATATTCTCTCATTCCTGAAAGAAGCAGTGTTGCTGTTTCTAATTTTTCCTTATCCTGTTTCCAGACTTGTCTTACAAAGTA**CACACAGATGTTAAAATTACTGG**GCTGGGAGAAGGGAGAGGGAGAAAATCCTACACTCTTACATGCACGCTGACTGAGG
27	mm4_intron_U6_17_68743603	GGGTAAATGAGGAGGGCTCGACCGAAGCACGTTAACTTCCCCGTGGCCTGAGCCGTTCGTCTGGAAGATTGAAAGCGCAAGTCCACAATTAGCCCATTAAGCCTTGGGACGCCGGGGTCTTGGAGATTTATTTATTTTTAATGCCAGACAAGAATGGCACTGAAAGAGCCATGAGGTGCAGGAGAAAATTAAAAAGCAGCAAGGAAGACTCGGCTGACGGCTGCAAGAAGCCCGTGGAGACGCCTACCTGCGGCCTCCTGTGGATTTTTTGCCCACTGGTGGGTCTGTGCCGCGGAAGGACTTCATCGGGGTCCACAGCCGCTGCGCTGACCCCTCTCCCCTCGGACACGCCCGTGGGCCTGTGGTCTGGGTCCCGCGGAGGACAGACGGGGCTGGCCTGGGGGCTGGCCGATCTGGGCGAGCATTGGGANAGGGG**CCTTTTGTTCTAGCGACTGTGGG**AAGCCAGGGCCTCCTCCTCGGTCGGTAGATAAATCTCCCTGGTGGTGCCCTGGGCAGCCGCAGAGCGCCGGGAGCTGTGACTGAAAAAGAAAATTTCTGATCATGCCTTCCTCTCGTGGCGGTCAAACAAAATGCCT
28	mm4_intergenic_ENSSSCG00000011811|TPRG1_13_135601574	TGGGAGGAGGATAGAGTGGTGGGAGACA**ACCACAGCCGCTTGAACTACAGG**TGCAGAATATGCCAGAAAGTTTGAGAAAAGAAGTCTAAATTCACTTGTTACACTGGTGATCCTGGGGAACTAGGGAGTTATCTTCCTTAGAAATGAGCCATTAAAATAATGTCAGACTTCTATATGATATTTGATTATTAAATAATCTGAAGTAACGTACTTTCACCATCAGTACAATGGAAAAAAGAGACTTGAATCTTTTATCTTTATAATCTCTTCCTTCTAGATTTCTAAATAACAGAAGACTTCTCTGTGCCTCTGACTGCTATCTGCTAGCCCCTCTGAAGCTAACGGCCACCACAGCCAGGCTCGCTGTGATGTGGACAGTGCCCACCAGAGGGACTAGAGGCACACTCACAGTTCACAGCCCGGGTTCCTCCCCTCTTCAGAGCTTCAAGATTGCTGTGGCTTCCACTCTTTATAAAATTCTTCCCTCACTCCAAAAAGTGAGGAAGTTTTCAACTAAATGTAGCTTCATCTTTATCTTACTCTATCCACAGAGCTAAACTTGAATGTGGTGCCTCATGTATCCC
29	mm4_intergenic_ENSSSCG00000025817|TTC14_13_127621134	AAGAGGTAGGAGGGGCGATTACATGATATAAGCAACCCAATACCTTCCAGGTGGGAAGC**CCCACAGACGAGAAAGCAACTGG**TTCACAGAGACTAATCTACAGGAGTGAGAGTTCTGAGCCCCATGTCAAGGCCCTATGCCTGAGCAACTGACATTGGGGAAATAGACCCTGGAGCACCTGATATTGAAGATCAGTGGGGCTTATGTTCAGGAGCTCCATGGGACTTTGGGAAATGGAGACCACATTCTTAAAAGATGCTCATAGACTTTGACGTGCACTGGGTCCCAGGGCAATGCAAAGTCTCCACTGGAATCTGGATCATATCTGGCTACATCTCTTGGAGATTCTCCTGGGAAAACAGGGGGTGAATGTGGCTTCCTGCAGGAGAAGGGCATTGGAAGCAAATCTCTCAGGAATACTCAGCAGCATTCCTTTCTCTGGAGGTGGCCATTTAAGGAAAATCTGGCCCCACCCATCAG<br>CACTGAGAAGC
30	mm4_intron_ENSSSCG00000015082_9_49999094	GCCTGGAAGAGACACCTGTCCTCTGGGGTGAGGCTGCTGTGGTTGGAGACAGAGCGGAGGCTGGGGAGCCAGGGAGCCTGGGCTTCTACCTCTCACCCCTCTTGGGGTCCTGGGTTTTTCCCATCCCTCCGGCATAGCCATGTCCCCGGGTCTCCAAGGCAGTGATGGCACAAGCATGGCCAGGAGTGGTCTTGTTGCAAGAGCTCCTCCAAGCTCAGGCTTTCCATGATGCCTGCTCTGGGAGTATCTCTTCATCCTTGAGGTGAAGGGCTGGCTCCATGCAGCTCTAAGGCAGACAGGTGTTTGTCTCTGTCTTCCTTCTGTGT**CCCACCGACTCTAAAATTCCAGG**GCTTTCTGGAGCCAGGCAGGAGGGAGATCCAAATCGCTCATTCCAGGAGGTGGGTGGACACATGGGAGGTTCGGGGTGTCTATCCACCGTCTGACCTTGACTCCAACGCCATCCTGGGAGGACCTCTTTCTGGCCCTTGGGTCTCAGTTTCACCGTCTGTGGGAGCCAGCTCCACAGAAGGACAG
31	mm4_intergenic_U6|ENSSSCG00000028308_1_45132160	TCTTCCTCCCCTGCTTCTCATCCTTTGCTCTGCTCTCAGTGATAGTTCTGTTACCAATGGTTTCTCTCTAGGCAGCAGTTCCTGTGATAGTTGTTTCCAGCGACAACCCTCTAAACATCTAATGCAATATCTAACACCCTCTAAAATCCTTAGTTTGGTTCTGGATCCCTTTGAAGGTGAAATACTACTGTGATCATCTCTGTAGGGCAGTTTATAACATTTATCACATTCCTTAACATTTGTTGAACACCTACAATATTGTAAACATTGCAGAAACAGTAATGAACACTCCGGTTGGAATCTCTGTAATAGGAAGCATGCGGTAAGGTAAGGAGAGGCACTTACCCCGTGAAGTTCTCTAGACAGTTGGGTTCAGTGTTCCCCTAGTAGCATCAGGAAGTTGACCTGTACATCTTTAGAGGCTTTCACAGCCTGGAAGAAGAGTACACTG**CCCTCGACTCCTTGTTGCTTAGA**GTAGGCTTCTATGCCTAGTCATGATGCCAGGATGCTCCACCTCAAGGTGTTCCAGAGGAAAGCCATTCACTCTCTCCTAGGTAGGTCCAAACCCAGGT
32	mm3_intergenic_ENSSSCG000	TGCTCCCTGGCTTTTCGATTTTTCG**CTCAAGCCAAAAGGAGCAGAGGG**CATCAAGTTGGGCATCCTTTCCAAGCGTGCTTGCTGGAGAGGGAACCTCTGGCAGCAGAGCGTCTCCCAGCTGCAACACGGACCTGGTCCTGCTGCCCGCT

	00022189|U6_9_103420505	GCCCACCGCGCCTGACAACCTCCACACCCACAAGAAACCGCCCGTGTGGCAGCAGTCATCGCACGGCTTCCTGTGCACAAAGCTTAAGGAGCCCCCTAATAGAAATAAATGTGTCCAGAGCCCAAAGCAGAGCTGGAGGTGGTTGGCTGATGACATGGGTCTTTCTGGGGGTCTGCGAGGACTAGGTAGCCCTTCACCTCTCGCACCTTGGCCGCAGATCTGTGCCTTGAAATCTGCCTCTCCAGAGACATAGACGCACCCACACAAACTCTCACTCACGCACACCCACCTCGCCTCTGNNNNNNNNNNNNNNNNNNNNGGCCGAACTGTAGGTACTGGCCCATGCTCT
33	mm4_intron_CCDC88B_2_6994077	GATCCTGTCACCACCACCAGACCTCCAGACTTTGGGGTTGGACCCCTTCTCAGGTGCTCTCTCCCACCACCCCTTCCTGCTTGTACCCCCTTCCCCTGC**CCGTTAACCCCTTGTGGCTTGTG**CCCTGCAGAGGAGGCGGTGGAGGAGCTAGAAGGCATCCTTAGGCTATTGCTGGGGGCGTCCGTGCAGGTGAGCGGGGGGAGGGAAGGGTACGGTCCTGCTTTGGCCGCAGAGGTGGGACACTCCGGCTCAGCCTCGTCCCCTCTTCCCCGCAGTGTGAGCACCGGGAACTCTTCATCCGACACATCCAGGGCCTCAGCCTCGACGTCCAGAGTGAGCTGGCTGCCGCCATCCAGGAGGTACACCTTTGGACTGGCACTGCCATGCAGGGACACCCTGTCCCCCACTTCGTTGGCTGAACTGCTTTCCCTGCGCCTCCAGGTGACCCAGCCCGGCACGGGCATGGTGCTGGCACTGGCTGGGCCTGACCCTGGCGAGCTGGCGCCCCCGGAGCTGGAGATGTTGTCCC
34	mm4_intergenic_ENSSSCG00000025823|ENSSSCG00000010368_14_95357154	AGGTTGTAGCCCCTTTTGGTTCTGATGTCTGC**CCCCCGACCCCTTGTGGCTGAAG**TTGGTATGGGGGCTTGCTGTAGGCTTCCTGATGGGACTGGTGCCTGCCCACTGGTAGGTGGAACTGATTCTTATCACTCTGTAGGTTGGGGTTTTGTCTCTGGGTGAGATTGGAGGCTGGCTGGGTGCCTGGGAGTACTTTAGGCAGCTTGTTTGCTGATGGGTGGGGCTGTGATCCCACCCAGATTATTGTTTGACCTGGGACTTCTTAGTCCTGATGGGTGGGGCCAGATTATTCCAAAATGGCCACCTGTAGAGGAGCACATGCTGATGATTATTTTCAAGACCTTTGCCTCCAATGTCCTTTACCCACAAGCCAGTCACCTGTTTTCCCAGGAGATTCTCCAAGAACTGCAATCGGGTCTGACCCAGATACCTATGGAGTCTCTGCTTTGCCCTCGGACCTAGCACACATGAAAGCCTGTGTGCACCTTTCAAG
35	mm4_intron_NR6A1_1_299084367	AGGGCATTGGAATTGGGTCTTAAAGGGCAGTAGGAGTTTCTTAGTTGAAGATTGGTGTTTCAAGGGTGGAAAGAATAGACTCAGGGACAAAGAGGAGTGTTGAGGCTTCTCTTGGAAC**CCAGAGGCTTCTTGTGGCTTGAG**AGGAAGGTAAATGGGCAAAAGGAGGGCCATGGTGAGCCATAGTGCTGGAAGACAAGCAACCAAGCTCCTCCAGAAACCTGGAGTATGGGGGTGGAGGGGAGGGAACTTGGGTTCTCTCCAGAGAGTTCGAGGAATCCCCATGAAGTTTAAATAAGTTCTCTGTTCCTGAAGGAGGAACAGAGGGTATCCTGAGCACCCAGTCTAACATGTATCAATTCCTTGTCAAAGATATCAGAGGAAGAGATCGAAAGGATTATGTCTGGGCAGGAATTTGAGGAAGAAGCCAATCACTGGAGCAACCATGGTGACAGCGACCACAGTTCCCCTGGGAACAGGGCTTCAGAGAGCAACCAGCCCTCACTGGGCTCCACACTGTCCTCCAGGTGAGCTTCAAGG
36	mm4_intergenic_SNORA70|5S_rRNA_11_58684299	CCTGGCTTCCCTTCCATCAGCAATTGCTCCCCTTATTTACAACTTCATATATTTGCCCAGACCTAAACCTCCTGCCCAGAGAAAGACCATGAGTTTCAGCCAGAGAACTGACACATCCAGCATTGATCCTTTAGTCTAT**TTCCAGCCACATGGAGTAGATGG**AGCAAAACTGGATAAGTATTAGCTTACAAGACTGTCTCATCCATTTAGTAGGAAAGAACCAGCAACTATTAATACTAGGTTCGTCTCACTATTTGCCATGAATTCAGGGAGTAATATTTAGAAATATGGTCATGGTATCATATTTAGAAATTGATTCTTTCACCAAAAGTTAAAAATGCAACCCAGGTCCTGGAACTACATGAAGGGGCAGCNNNNNNNNNNNNNNNNNNNNTGGCCCACTTTCCTCAAGAGTTCAAGAACACACATAAAAAAATGTTAGTATATATTCATTCAATCCTTTCAGTCTCCTATCACCCAGTTGAGTAAGCAAGT
37	mm4_intron_HOGA1_14_118469185	CTTCACCGGGAGTCCTTCACCAGCAAGCAGAGGATGTCTGGGACCTTGGTGACCAAAGAAACTCCCAAGGAGTCTGAAAAATCCTCGGCCACGGAGGAGGCCACGTGGGCCGCCGTGGCTGCCTGTACCAAGGACATTGACGCCAAGGGACAGCAGCTGGCCAACTCCATGCTGCAGCGTGTCATGGCGTGCCAGAA**CTCAGGCCACCTGGAGTCCAAGG**ATATCAACCAGGAAGAGCTGAAAGCGCTCGAGGAGGTGCAGTTAAAGCTGAAAGGCAATTTCTTCACCCAGCGGGAAGCCACCATAGCTGGCGCCAACCACACGCACACCTTCCATAGTNNNNNNNNNNNNNNNNNNNNNNNNNNNNNNNNNNNNNNNNNNNNNNNNNNAGCCTGCCCAACCGCAGCCATTAGACCACCTAACCACGGATGGACATGCCCCTTCCCCCAACAAGGCTGGCACACACAGGCCTGTTTTCTCTCCGATGGGGCA
38	mm4_intron_TMEM182_3_53891758	CATTCATGCTGCCTTCCACGGGAAGCCTCCCCATCTACTAAGAAGGCACTGGCTATGTTTTACACCACCTGCCTCCCAAATACCCAAATTCCAAACAAACAGGGTCAGAGACCAGACACAAAGAACCAGAACACGGTAGCCTCTGAAGTGGGGCAAAGACTCACTGAGCTAATCAGTGCCACCCCCTGAGAACAGGAACACTGGGGAAGTGAGGGGATGTTGGGTAGAG**CTGAAGCCACAAGGAAGCAATGG**TAGCTGATCCAGAGGAACCTGGACCGTGCACAATCAGAGGTGGGAATTAGGACTCAGGCCACAGAGAGGGCAGAAAAAGCAGAGGAGGCTCTCTGCACAGAGAGAAGCAGAGGGACGGCAAGAAAGAGAAAAACCAAGTGAGGGGGAGCGAGAATAGAGAACCAGACAGAAATGGGGGATAAAGTTTCCCCGGAACTTAAATCCTCGGACCTGCTATTTCCCGCCCTTGGTTTACTTC
39	mm4_intron_LGALS8_14_58861814	CCAATGTGGACCAAAACTTCACAAACATTAAGAAATTAAAATAAAAAGGTAGCAGTTCTGTCTTCATAAGGCTTAGTAAAACTATAGTAGAAACTCAACACTTCCTTGTTGTTAGAACTGATCACTAAGAAAATAGCCACAATGCNNNNNNNNNNNTTTACTCTGGAAATGTGGTATCATGACCTGTCCCCAGCGCAGCAGAGGCCACTGGGAGCCCTAAGTGCAGCTGCTGGTCTAAAGTAGCACTACCCCTCAGTTCCCCTTTCTCGGGAAATGAAGCGCTGGCTTATTTTTGTTTCAAGTCTGTAGCTGAAGGTGCTAACCCCGGAAATTACCAGTTCATCATTATACAGGGATATGCAGGTATTTAATCTATAAAATTATTTTCTAATAACATTCAAATAAGGCATCTCTGTGATGAAGACCAGAACTGAAAGTACAACCTGA**CCATCCATTCCTCCTGGCTTGAG**CCAAGATTACACTTCAGTAACCCTTGACAGGCAGC
40	mm4_intergenic_NAMPT|CCDC71L_9_116980314	TCGGTTCGAGAAAAGCTCCCAGAGAAGCAGCTCTCCTTCAAGGGACTTCCTGCAACTACACAGTCTTTCTCATTTACTTTGTGCAAAAAGACCAACTCTTCTACCAACCAGCCTTCATGTGAAGGCTCTCTTTCTACAGAGCTCTTGCATATGTCAATTACCTTTCTATCCCCCAAGCAACTCTTCCCAAAGGCTCGCTGGCAGCTGAGGCTCTATAGATGTCTACTGAGCCAGCATGTGGATGTGAAACAAGATGC**CACATGCCACAAGGAGGTGAGGG**CACCAAATAATTCACACTTCTCTGTTCTCAAGAAACTGATAGGCTCATGCAGTGATATGAAACTATGGGAAAGCAAATCCAACTAGTATTTGAACTGAGAACCCAAGTTATTTATATGCTAAAGAAAGCAGGGAGCCCTGGGGGCTAGAGTCCTCAGGGATGCTTCACACAAGAGGAGAAAGCTGGAGGATGGAGGGGGTTCTGGACGGTGGAGAGGCACCAGGGATGAGGGGAGCCAGCAGGAGGGTTAGAGGGCAGGAGAG
41	mm3_intergenic_KIT|U6_8_43856928	GTACCACCAAGCTCCTTCCCCAAGAACTACATTCAGCACCCCTCTATCCAGCCACCATCACTTTGAATTGTGTCTGAGCATTTGTGCTTTATCTCTATTGATTTGGTGCATCTGTTAGCACGACATGTCTTAGGATAATTGCTTCTTCACTTTATGCCATTTCAGCTCAGGAAAAATTTCTTAAGAACCACTATTTGTGGATAGTGTGGGGTGTAGTTTTGAACATGCCAATAAAATGGTTATACCATTTACAAAGGATTAGAGGAATCCACACAATTCTCCAGGCCAAGGATTATCCATAGGCTATGAAGTTCAATCAATGACCCCAGTGGGTAAATAAAAGAGAAATTCGAGATCCCAAA**CCTCAAGGAGACGAATGAGACAG**ATGAGACCTTTATGGGTGGACAGAGAAGCAGAAGAAGCTTCTCCATTCATGACGCTCAGAAAAACAAAGTTGTGTCAGACAAGATTGAACTACCCAAAGATCTGACTGCAGTTTCCTGGGGCCCCCTAAAAAGGAGGTGGGAGCATGTTTTGGG
42	mm4_intron_ENSSSCG00000008043_3_42355845	CGTGGCATCAGACAAAGCTGGAACTGGGCAGTGGGGCTGGGGCTGAAGTCACTGTGCCCACCTGGCCCTCCGCCGCCCTGTGCAGGAGGGGGCGGGCTGTCTGGATGCCTTTCAGGGGCTGAATGATCCAGGGAGCACCTAGGGAGGGTACCTGGGTGGGAGGAGCAGCCTGGCCAAGACTAGGACCTGAGGGTGAGGGCGTGGTGGTGCCACAGCAGGGGCTGGAGGGTGTGGCTGGGCGAGGAGGGGCCCTGGTCCAGGGACTGGGCCTGCACTCCCCGACCT**CCACAGGGAATCGCAGGAGACAG**AAGGCACAGAGGAGTGATGAGAAAACGGCCAAGGGCGCACGGAGGTACAGACTGGGGCCTGCATTGCCAGCCACTGGGCTCTCGGGTGAAGGCCCAGGTCCTGTCTCCATGGGTTCAGACTCACGGATTTAGGACAGCCAGGGACTGGCTTTCCAGTTCCTCCCCTGTAGTCGGGGCAAGGCTGGGCACTGAGTTGGAGGGAGGGTTTGGGCAGGAAGACGTGGCCCTGCAGCTGGGCGCTGAGATCGGAGGATGGATGCTGCCTCTTCCAGGACATT
43	mm3_intergenic_SLC13A4|ENSSSCG00000026	CAGAAGGTTCCTCCGTCCACCGCCTCGAGTGGAGCGCAGGGAGTCTCCCACGAGAGCACAGGCTCGGGTGCTGGTGTCTCCTGCTCCGCCCTCCCTCCTCAGCACCTTGGCTCGTGGAGACGTAAAGCCTTCCCTTCCCTCCCCATATATCGTCTCTCCAATCAGCTCTTCTGAAAACAGGACGAGACAAAAGCCTGTCTTGGTTGCCCTCAGAGGCTGAGCCTCAACGACTGAAGAGTCTCACTGGGTTTGATGTTATTCTTAATTTTGTAATCC**CCTTTAGGAGTCGAAGGAGAAAT**C

	054_18_14367787	CATCCTGCCTTCACCCAGAGAGCAAAACGAGTGACGTCTCCTCTTTGGGAGAAAGTCTCACCCAGCACTTGAGCC<br>TAACATCAGCTCCTTTCTCCTAAGGGTTTCTGTCCTGAGTTCTCTCCACACAAAGGCAGTTCTTACGAAGGCCTCT<br>CTGTGGCTATCAGCACTCTTATCATTACTTCAAGTCCCTTCTAAGCATCTTTCTTCAATAGCTTCTCTATGCCTTGC<br>ACCAAATTCCTGTAAAGAAATAGTGTTTTCCTTCAGGAAAGAGGCTGGCTG
44	mm4_intergenic_U6|ENSSSCG00000021497_10_78541669	AGCCATCTGGGATCCTCTGTTTTCATTTTGATAGTTTAGCTTCATAGATTCCTCTAGAAGACAGCTTGTTCTGTTCA<br>CATTTCTCCTGGAAGAGTCCATGGTCTTGAGAGCCGTATTCTTTACATATTGTAGCTGCAGCAAAGATTGAACAG<br>GGCCCCTCAAATCATGAACTGTGGGCTTGGGAACATCTCTTAGGGCTGTGATCATGGAAATTTTGCCTCCTCACTA<br>TTCCTACTGTGCA**ATTTTTCCTTCCATTCCTTGTGG**AAAAATGTATATTCTAGCAACAATCATTTCAAAAGTAAT<br>AGAAATTGCTGTTTCAAATTTGCATTATATATAATATTCAAAATGACATGAGGNNNNNNNNNNNNNNNNNNNN<br>NNNNNNNNNNNNNNNNNTTTCAATGCTAAATTAATAGATTAAAACCCCATTCTAATTACCCTCCTTGTGAAATTCA<br>TTGAATCTACTGAATTTTTGGCCCAGAATTTTTATTAAAGTCACCAATAAAAGATTTAGTCTTCATCTGTCTAGCT<br>AAGTATATTCCTTTCGTTAAAAATCTGAGAAACATGTCCCCTTCACATTTCAGGTCTTCTCCTCTCCACT
45	mm4_intergenic_GORAB|METTL11B_4_88175014	CTTAGGCTTCTCCCGCTTGGAATCCCGATTGCAATGTACTAGTAAGACCTATCAATTTCTCATTTTAAGCAATATT<br>TCAGAGATTTAAAAATGCGAGCGTTGTTAAAGTACATATAGTCAGGATGATTTAAAAGAAACCACTTATTTAATT<br>TTTCAGATTTCTCTTCTCACTGTTTTGCATTTTAAAACACAGTTATTTTGGCTTACATTTAAAAATCCCATGGGTGG<br>CATTTGGCTTTAGTGTGCCAATATTTTAAAACAGAATAAAGGAGTAGGCTAAAAATAAAATACTTACTAAGAAAC<br>GATTTGTAATGAGTATTTGAGACTTAAATAGGTGATTAGAAGATGGGATGTTTGGAATTCTCTTTGTAACTTTTAA<br>GCTTTTATACTCTAAAAGGATTTTTGAAATATAGGTGTCCACAAACACTTCATTTTAAATGTGGACTCATTTTCCC<br>TAGCATTTAAAAGTTAAAATGTCAT**CCAAAAGGATTGGAAGGAGACAG**CTTCATCCAGTTTTTGAACTTAAACT<br>NNNNNNNNNNNNNTTAAGATTATCTAGCTCCGAAATAATAGATGGGGA
46	mm4_intergenic_RNF19B|ENSSSCG00000030312_6_83284851	GACCCTCAGTGCCTGAACTCTTCTCTTTAGGAAAGAGGATGGCTTTCACTGGGAAAATACTCTGAAAAACCTTTT<br>AAGCTGAAGGTGTGATCTTGAAGCTCCCAGAGGCAGCTGCGATGGAGGCAACAAAGAAGAGGATTTGGGATTTG<br>AATTTGTTCCTAAAATGCCACAAATGTTTTAAGCTACCAAGGACTCAGAGAATCTACCAGAAACTGGAGGATTGA<br>ATTTGGGAAATGGGCAGGAACCAAGGGTAGGGGTGGAGTTGGGGGTGGGACAGAAGGGAGGCAGCAGGAAGCC<br>AGCCAGGGACACCCACCTACTCCCTACTCAA**CCACTAGGAATGGTAGGAGACAT**TTCCTCCCATCCTCATGGGG<br>GCTACATGAAGGCCGGAATAGGCTTGGACTTGCAGGTGGTGAGGGTGAGGGGGTGACTGTGACCAGGAGACAGG<br>AGAGGAAGCCAACCCCTCCAACCCCTAAGGCTTCATGCAGTGGGG
47	mm4_exon_LST1_7_27539143	CGATGTGCCTCTTTCTGGGTTCTCCCGCCTCTTGCGCTCCCTCTACCACCTCTGGCCTGGAAAGGTCCAGGGAGGG<br>AGCTTCCCAGCACCTACGCCCACCCACCACCTCCCTCTCCCATGCCCGGGAGGCTAAGGCCAAGTGGAGTATCAT<br>TTGTGATTGATCCTAGTGGCCAACGGCCTGCAGACCAATGTCCTTCTTTCTGGCCCTCAGAGGAGCTTGAGCCTCT<br>AAACACCCATCTTCACCCAATGACTCCCTACTTGACCCCAGAAATGGGCGCCAGGGTCAGTGGCTCAGGTTTTGG<br>TGGTGGCCAAAACCTAAGCTGGGGCTTGAGATGGACGGAGCAGCCTGAGACACAGAAGGTGGTAATAGTCTTTA<br>CTGGGGGGACCATAGGGCAGCCCTGACTACCCGCTCGGGTTAGGGGAGGCGGCTGGGGGGCTCAGGTGGGCTTG<br>TCCTTGGCAATGCAGGCGTAGTCCGTACTGAGATCCTCC**TTGTCTCCTTCCCTTCCTTGGGG**AGGGTGCTCCCG<br>TACTGGCAGCCTCAGCAGGGTTGCATAGTAGGGCTCC
48	mm4_intron_ENSSSCG00000021813_9_118320042	ATCAACACCACAGACCTGCCCTTCCAGATAGACTGGAATGCTGATCTTCCTGTCAACATCGTGGTCCCTAAAATC<br>AGCCTGCATAGCCTCATTCTTGACTTTTCAGCAGTGTCATTTCTTGACGTTTCTTCCATGAGAGGGCTCAAAACGG<br>TAATTTCTCTTTTCTTAAAGGAAACAACCAATTGGTGAGTGAAGGTAAAAAAACGTTGTCTTTGTGGAAGGATGC<br>TCCTTGAGAGAACACACACTTAAGCAGAAGTAAATTTTAATCTTAGAAGATTTCAGGGGATAGTGCCTCTGGGGT<br>ATGTTCAAAGAATTCTTTAAAAGTGCATTTTCTCATGCAGCTACATGTGATTTTGAAGGCAACCAAACTGACATTT<br>CTAATGTATGCTGAGCCTACAGGTAGACAACAATGCCCACACCCCTGTCAAATTCT**ATGGTGCCTTCGAATCCT**<br>**TGTGG**GCCTTGCATCTAAATTGTCCTGACATAGCAACGATTTGGAGAGGTCT
49	mm4_intron_ENSSSCG00000015700_15_20000512	CCCAAGCATGCATTTGCCAAGTTTGAGTTTTGTTTGTTTGTTTGTCACAAACCTCTTTTATTGCCGCTAGGATCAAT<br>GAGAAAGGATCTTTCCTCTTAGTGATCTCAGAGAGGGAGACAATGAAGGAAGAAAGTGTCACAGAACTGCGAGA<br>ACGTGGTTCAGGAAGCATGGGTGAAGTGTCAGTTCTTGGAGTATGAGTTGTCAGGAAGCACTTGAGACAAGTCTA<br>TAAAAGGGATTGTTTACTCGTTCAGGCAGTGGAAACACAGCAGTAGAGGTAATTCATGAGATCTGAAGGCAGCG<br>CATGCCGAGTTCATGTTTCAGCCCCTGACCTCTTAAACCTCTGATCCAATCTTTGTAATGACTATGATTATGATCT<br>CTGTCCTCTACCTCACAGTGTGACAGTGGACGTTATGCCAGTTATTATAATGTACAGAAGATGAATTATATAAAT<br>ATTAGTAG**TTGTCACCTTGGAATCCTTGAGG**TGAATTGCTTTTTAAAAAAATATTAGCTTTTCCCACCCCTACT
50	mm4_intron_ENSSSCG00000004369_1_81580139	TGAAGACTGGCACCTGAAGCCAAATCTCCCTACTTGCCCTTGAGGCCTGGTATCCCTGGGAGAAACTCTTAAGTA<br>TCAGTAAATAATTGATCACTTAATTCACTTCTTTCATTGCTTTCTCTTGCCCTCAGTGGGCCCCTCCTCATTTTCTC<br>CAGCCCGCCACTGTTCCATACTTATTAGTAAACAGTTAAATCTTTTGGCAAGTTGACACGTCCTGGAAGAGCTAA<br>GTGGCAGCAAGGGGTGAGTGAGATTGTCAGCAACTTGGAGAAAATATCTGGCCACTCTGGACTCACCGGCCCTT<br>GACCTCTCATTGTACAATAGCTTTTGAGGGAGAAAACTTTTTGGTAGGGGGGAAAAAAATGTTTTTC**ATGTTTCC**<br>**TTTGTCTCCTTTTGG**TCATGAGATGGCTTTATTCTTTTTTAACGAGCCGGCTTTATTAGCTGGGTTTCTAAAATTA<br>TTCTCAAAACCTTGACGTGTTTATGAACTGATGGTATAAATGGAGAAGAGGTTCGTGTAAAAGTGTCCTCTGTCC<br>GCATGACTTCAGCGCTAAC

Note: Primers underlined, off-target sites in bold. Replace ambiguous bases with N. The potential off-target sites corresponding to tyr-sg1, tyr-sg2, il2rg-sg1, rag1-sg1, and rag1-sg2 were 1-10, 11-20, 21-30, 31-40, and 41-50, respectively.

Table S4. *TYR* mutation of F_0 generation piglets

Piglet ID	Sex	Genotypes	Indel
		GTTCCCCTTCACCGGGGTGGATGAACGGGAG//TGCCAGTGCTTTGGCAACTTCATGGG	WT
a1	Male	GTTCCCCTTCACCGGGGTGGATGAACGGGAG//TGCCAGTGCTTTGGCAACTTCATGGG	allele 1: △WT
a2	Male	GTTCCCCTTCACCGGGGTGGATGAACGGGAG//TGCCAGTGCTTTGGCA------TCGACTC<br>GTTCCCCTTCACCGGGGTGGATGAACGGGAG//TGCCGGTGCTTTGGCA------CCTTGGC	allele 1: △352<br>allele 2: A→G,△352
a3	Female	GTTCCCCTTCAACCGGGGTGGATGAACGGGAG//TGCCAGTGCTT-------------------TGGG<br>--TCCCC-----------------GGGGTGGATGAACGGGAG//TGCCAGTGCTTTGGCAA--------GGG<br>GTTCCCCTTCA---CATGGG	allele 1: +1, △11<br>allele 2: △14<br>allele 3: △70
a4	Female	GTTCCCCTTC--CGGGGTGGATGAACGGGAG//TGCCAGTGCTTTGGCAA---------A--GGG<br>GTTCCCCTTCA---CATGGG	allele 1: △7<br>allele 2: △70
b5	Female	GTTCCCCTTCACCGGGGTGGATGAACGGGAG//TGCTTTGGC-----------------GACTTTTGG<br>AGGCACCCC---TGGG	allele 1: △67<br>allele 2: △93
b6	Male	GTTCCCCTTCAGTTCATCTCCCCGGGGTGGAT//CAGTGCTTTGGCAACTTCTCCATGGG<br>GTTCCC--CTTCATGGG	allele 1: +10, +3<br>allele 2: △72
b7	Female	GTTCATCCC--------CCGGGGTGGATGAACGGG//TGCCAGTGCTTTGGCAAC------ATGGG<br>GTTCCC------------GGGGTGGATGAACGGGAG//TGCCAGTGCTTTGGCAACT-------TGGG	allele 1: +2, △7<br>allele 2: △10
b8	Female	GTTCCCCT---------GGGGTGGATGAACGGGAG//TGCCAGTGCTTTGGCAA-------CATGGG<br>GTTCCCCT---------GGGGTGGATGAACGGGAG//TGCCAGTGCTTTGGCAACTT-------GGG<br>GTTCCCCTTCACCGGGGTGGATG---CATGGG<br>AGGGGTTCCT---CATGGG	allele 1: △8<br>allele 2: △8<br>allele 3: △58<br>allele 4: △115
c9	Male	GTTCCCCTTCACCGGGGTGGATGAACGGGAG//TGCCAGTGCTTTGGCAAC------ATGGG	allele 1: △3
c10	Male	GTTCCCCTTCACCGGGGTGGATGAACGGGAG//TGCCAGTGCTTTGGCAACTTCATGGG<br>GTTCCCCTTCACCGGGGTGGATGAACGGGAG//TGCCAGTGCTTTGGCAACT------TGGG<br>GTTCCCCTTCACCGGGGCGGATGAACGGGAG//TGCCAGTGCTTTGGCAACT------TGGG	allele 1: WT<br>allele 2: △3<br>allele 3: T→C, △3
c11	Male	GTTCCCCTTCACCGGGGTGGATGAACGGGAG//TGCCAGTGCTTTGGCAACTTCATGGG<br>GTTCCCCTTCACCGGGGTGGATGAACGGGAG//TGCCAGTGCTTTGGCAACTTTCATGG<br>GTTCCCCTTCACCGGGGTGGATGAACGGGAG//TGCCAGTGCTTTGGCAAC------ATGGG<br>GTTCCCCTCACCGGGGTGGATGAACGGGAG//TGCCAGTGCTTTGGCAAC------ATGGG<br>GTTCCC--CTTCATGGG	allele 1: WT<br>allele 2: +1<br>allele 3: △3<br>allele 4: T→C, △3<br>allele 5: △72
c12	Female	GTTCCCCTTCACCGGGGTGGATGAACGGGAG//TGCCAGTGCTTTGGCAACT------TGGG	allele 1: △3
d13	Male	GTTCCCCTTCACCGGGGTGGATGAACGGGAG//TGCCAGTGCTTTGGCAACTTCATGGG<br>GTTCCCCTTCACCGGGGTGGATGAACGGGAG//TGCCAGTGCTTTGGCAACT----ATGGG	allele 1: WT<br>allele 2: △2
d14	Male	GTTCCCCTTCACCGGGGTGGATGAACGGGAG//TGCCAGTGCTT---------------------TGGG<br>CCCTGGGGAG--TTGTAAGTTT	allele 1: △11<br>allele 2: △184
d15	Male	GTTCCCCTTCACCGGGGTGGATGAACGGGAG//TGCCAGTGCTT----------------------TGGG	allele 1: △11
d16	Female	GTTCCCCTTCACCGGGGTGGATGAACGGGAG//TGCCAGTGCTT----------------------TGGG	allele 1: △11

Note: Underlines mark target sites. Red text indicates base insertion; yellow highlighting indicates base mutation; (-) indicates base deletion. (+) denotes the number of inserted bases; (△) denotes the number of deleted bases; (→) represents the mutant base.

Table S5. *IL2RG* mutation of F_0 generation piglets

Piglet ID	Sex	Genotypes	Indel
		GAACCCAGGAGGCAGGACCCACAGACGCTAAAACTACAGGATCTGGGTAATTTGGAAAT	WT
c9	Male	GAACCCAGGAGGCAGGACCCACAGACGCT------------------------------GGGTAATTTGGAAAT	allele 1: △15
c10	Male	GAACCCAGGAGGCAGGACCCACAGACGCTAAAACTACAGGATCTGGGTAATTTGGAAAT<br>GAACCCAGGAGGCAGGACCCACGGACGCTAAAACTACAGGATCTGGGTAATTTGGAAAT	allele 1: WT<br>allele 2: A→G
c11	Male	GAACCCAGGAGGCAGGACCCACAGACGCTAAAACTACAGGATCTGGGTAATTTGGAAAT	allele 1: WT
c12	Female	GAACCCAGGAGGCAGGACCCACAGACGCTAAAACTACAGGATCTGGGTAATTTGGAAAT<br>GAACCCAGGAGGCAGGACCCACGGACGCTAAAACTACAGGATCTGGGTAATTTGGAAAT<br>GAACCCAGGAGGCAGGACCCACAGACGCTAAAAACTACAGGATCTGGGTAATTTGGAAAT	allele 1: WT<br>allele 2: A→G<br>allele 3: +1

Note: Underlines mark target sites. Red text indicates base insertion; yellow highlighting indicates base mutation; (-) indicates base deletion. (+) denotes the number of inserted bases; (△) denotes the number of deleted bases; (→) represents the mutant base.

Table S6. *RAG1* mutation of F_0 generation piglets

Piglet ID	Sex	Genotypes	Indel
		ATATAATCACCATATCTCAAGCCACAAGGAGTCGAAGGAGACATTTGTGCATATTAAT	WT
d13	Male	ATATAATCACCATATCTCAAGCCACAAGGAGTTCGAAGGAGACATTTGTGCATATTAAT<br>ATATAATCACCATATCTCAAGCCACAAGGAGTTCGAAGGAGACATATGTGCATATTAAT	allele 1: +1<br>allele 2: T→A, +1
d14	Male	ATATAATCACCATATCTCAAGCCACAAGGAGTTCGAAGGAGACATTTGTGCATATTAAT<br>ATATAATCACCATATCTCAAGCCACAAAGGAGTCGAAGGAGACATTTGTGCATATTAAT	allele 1: +1<br>allele 2: +1
d15	Male	ATATAATCACCATATCTCAAGCCACAAGGAGTTCGAAGGAGACATTTGTGCATATTAAT<br>ATATAATCACCATATCTCAAGCCACAAGGAGTGTCGAAGGAGACATTTGTGCATATTAAT	allele 1: +1<br>allele 2: +2
d16	Female	ATATAATCACCATATCTCAAGCCAC-------------------AAGGAGACATTTGTGCATATTAAT	allele 1: △9

Note: Underlines mark target sites. Red text indicates base insertion; yellow highlighting indicates base mutation; (-) indicates base deletion. (+) denotes the number of inserted bases; (△) denotes the number of deleted bases; (→) represents the mutant base.

Table S7. *IL2RG* mutation of F_1 generation piglets

Piglet ID	Sex	Genotypes	Indel
		GGAACCCAGGAGGCAGGACCCACAGACGCTAAAACTACAGGATCTGGGTAATTTGGAAAT	WT
1	Male	GGAACCCAGGAGGCAGGACCCACAGACGCTAAAACTACAGGATCTGGGTAATTTGGAAAT	allele 1: WT
2	Male	GGAACCCAGGAGGCAGGACCCACAGACGCTAAAACTACAGGATCTGGGTAATTTGGAAAT	allele 1: WT
3	Female	GGAACCCAGGAGGCAGGACCCACAGACGCTAAAACTACAGGATCTGGGTAATTTGGAAAT<br>GGAACCCAGGAGGCAGGACCCACAGACGCTAAAA------CAGGATCTGGGTAATTTGGAAAT	allele 1: WT<br>allele 2: △3
4	Female	GGAACCCAGGAGGCAGGACCCACAGACGCTAAAACTACAGGATCTGGGTAATTTGGAAAT<br>GGAACCCAGGAGGCAGGACCCACAGACGCTAAAA------CAGGATCTGGGTAATTTGGAAAT	allele 1: WT<br>allele 2: △3
5	Female	GGAACCCAGGAGGCAGGACCCACAGACGCTAAAACTACAGGATCTGGGTAATTTGGAAAT<br>GGAACCCAGGAGGCAGGACCCACAGACGCTAAAAACTACAGGATCTGGGTAATTTGGAAAT	allele 1: WT<br>allele 2: +1
6	Female	GGAACCCAGGAGGCAGGACCCACAGACGCTAAAACTACAGGATCTGGGTAATTTGGAAAT	allele 1: WT
30	Male	GGAACCCAGGAGGCAGGACCCACAGACGCTAAAAACTACAGGATCTGGGTAATTTGGAAAT	allele 1: +1
31	Male	GGAACCCAGGAGGCAGGACCCACAGACGCTAAAAACTACAGGATCTGGGTAATTTGGAAAT	allele 1: +1
32	Female	GGAACCCAGGAGGCAGGACCCACAGACGCTAAAACTACAGGATCTGGGTAATTTGGAAAT<br>GGAACCCAGGAGGCAGGACCCACAGACGCTAAAAACTACAGGATCTGGGTAATTTGGAAAT	allele 1: WT<br>allele 2: +1
33	Female	GGAACCCAGGAGGCAGGACCCACAGACGCTAAAACTACAGGATCTGGGTAATTTGGAAAT<br>GGAACCCAGGAGGCAGGACCCACAGACGCTAAAACTACAGGGTCTGGGTAATTTGGAAAT	allele 1: WT<br>allele 1: A→G
34	Female	GGAACCCAGGAGGCAGGACCCACAGACGCTAAAACTACAGGATCTGGGTAATTTGGAAAT	allele 1: WT
35	Female	GGAACCCAGGAGGCAGGACCCACAGACGCTAAAACTACAGGATCTGGGTAATTTGGAAAT	allele 1: WT
36	Female	GGAACCCAGGAGGCAGGACCCACAGACGCTAAAACTACAGGATCTGGGTAATTTGGAAAT	allele 1: WT

Note: Underlines mark target sites. Red text indicates base insertion; yellow highlighting indicates base mutation; (-) indicates base deletion. (+) denotes the number of inserted bases; (△) denotes the number of deleted bases; (→) represents the mutant base.

Table S8. *TYR* mutation of F_1 generation piglets

Piglet ID	Sex	Genotypes	Indel
		GTTCCCCTTCACCGGGGTGGATGAACGGGAG//TGCCAGTGCTTTGGCAACTTCATGGG	WT
1	Male	GTTCCCCTTCACCGGGGTGGATGAACGGGAG//TGCCAGTGCTTTGGCA------------TGGG<br>GTTCCCCTTCACCGGGGTGGATGAACGGGAG//TGCCAGTGCTTTGGCA------------TGAG	allele 1: △6<br>allele 2: △6, G→A
2	Male	GTTCCCCTTCACCGGGGTGGATGAACGGGAG//TGCCAGTGCTTTGGCA------------TGGG	allele 1: △6
3	Female	GTTCCCCTTCACCGGGGTGGATGAACGGGAG//TGCCAGTGCTTTGGCA------------TGGG	allele 1: △6
4	Female	GTTCCCCTTCACCGGGGTGGATGAACGGGAG//TGCCAGTGCTTTGGCAAC------ATGGG	allele 1: △3
5	Female	GTTCCC---CTTCATGGG	allele 1: △72
6	Female	GTTCCC---CTTCATGGG	allele 1: △72
7	Female	GTTCCC---CTTCATGGG	allele 1: △72
8	Male	GTTCCCCTTCAGTTCATCTCCCCGGGGTGGAT//CAGTGCTTTGGCAACTTCTCCATGGG<br>GTTCATCCC--------CCGGGGTGGATGAACGGG//TGCCAGTGCTTTGGCAAC------ATGGG	allele 1: +10, +3<br>allele 2: +2, △7
9	Female	GTTCCC---CTTCATGGG<br>GTTCATCCC--------CCGGGGTGGATGAACGGG//TGCCAGTGCTTTGGCAAC------ATGGG	allele 1: △72<br>allele 2: +2, △7
10	Male	GTTCCCCTTCAGTTCATCTCCCCGGGGTGGAT//CAGTGCTTTGGCAACTTCTCCATGGG	allele 1: +10, +3
11	Female	GTTCCC---CTTCATGGG<br>GTTCATCCC--------CCGGGGTGGATGAACGGG//TGCCAGTGCTTTGGCAAC------ATGGG	allele 1: △72<br>allele 2: +2, △7
12	Female	GTTCCCCTTCAGTTCATCTCCCCGGGGTGGAT//CAGTGCTTTGGCAACTTCTCCATGGG<br>GTTCATCCC----CCGGGGTGGATGAACGGGAG//TGCCAGTGCTTTGGCAAC------ATGGG	allele 1: +10, +3<br>allele 2: +2, △7
13	Male	GTTCCCCTTCAGTTCATCTCCCCGGGGTGGAT//CAGTGCTTTGGCAACTTCTCCATGGG<br>GTTCCCCTTCACCGGGGTGGATG---CATGGG	allele 1: +10, +3<br>allele 2: △58
14	Female	GTTCCC---CTTCATGGG<br>GTTCCCCTTCA---ACCTCATGGG	allele 1: △72<br>allele 2: T→C, △66
15	Male	GTTCCCCTTCAGTTCATCTCCCCGGGGTGGAT//CAGTGCTTTGGCAACTTCTCCATGGG<br>AGGGGTTCCT---CATGGG	allele 1: +10, +3<br>allele 2: △115
16	Female	GTTCCC---CTTCATGGG<br>GTTCCCCTTCACCGGGGTGGATG---CATGGG	allele 1: △72<br>allele 2: △58
17	Female	GTTCCC---CTTCATGGG<br>AGGGGTTCCT---CATGGG	allele 1: △72<br>allele 2: △115
18	Female	GTTCCC---CTTCATGGG<br>GTTCCCCTTCACCGGGGTGGATG---CATGGG	allele 1: △72<br>allele 2: △58
19	Female	GTTCCCCTTCAGTTCATCTCCCCGGGGTGGAT//CAGTGCTTTGGCAACTTCTCCATGGG<br>GTTCCCCTTCAACCGGGGTGGATGAACGGGAG//TGCCAGTGCTT--------------------TGGG	allele 1: +10, +3<br>allele 2: +1, △11
20	Female	GTTCCC---CTTCATGGG<br>GTTCCCCTTCAACCGGGGTGGATGAACGGGAG//TGCCAGTGCTT--------------------TGGG	allele 1: △72<br>allele 2: +1, △11
21	Male	GTTCCCCTTCAGTTCATCTCCCCGGGGTGGAT//CAGTGCTTTGGCAACTTCTCCATGGG<br>GTTCCCCTTCAACCGGGGTGGATGAACGGGAG//TGCCAGTGCTT--------------------TGGG	allele 1: +10, +3<br>allele 2: +1, △11
22	Male	GTTCCC---CTTCATGGG<br>GTTCCCCTTCAACCGGGGTGGATGAACGGGAG//TGCCAGTGCTT--------------------TGGG	allele 1: △72<br>allele 2: +1, △11
23	Female	GTTCCC---CTTCATGGG<br>CCCTGGGACC------------CCT--------GGGGT//TGCCAGTGCTTTGGCAA-----------------TGGG	allele 1: △72<br>allele 2: △18
24	Female	GTTCCCCTTCAGTTCATCTCCCCGGGGTGGAT//CAGTGCTTTGGCAACTTCTCCATGGG<br>GTTCCCCTTCTACCGGGGTGGATGAACGGGAG//TGCCAGTGCTT--------------------TGGG	allele 1: +10, +3<br>allele 2: +1, △11
25	Male	GTTCCCCTTCAGTTCATCTCCCCGGGGTGGAT//CAGTGCTTTGGCAACTTCTCCATGGG	allele 1: +10, +3
26	Female	GTTCCC---CTTCATGGG<br>AGGCACCCC---TGGG	allele 1: △72<br>allele 2: △93
27	Female	GTTCCCCTTCAGTTCATCTCCCCGGGGTGGAT//CAGTGCTTTGGCAACTTCTCCATGGG<br>AGGCACCCC---TGGG	allele 1: +10, +3<br>allele 2: △93
28	Female	GTTCCCCTTCAGTTCATCTCCCCGGGGTGGAT//CAGTGCTTTGGCAACTTCTCCATGGG<br>GTTCCCCTTCATCCCCGGGGTGGATGAACGG//TGCCAGTGCTTT-------------------------GG	allele 1: +10, +3<br>allele 2: +3, △12
29	Female	GTTCCC---CTTCATGGG<br>AGGCACCCC---TGGG	allele 1: △72<br>allele 2: △93
30	Male	GTTCCC---CTTCATGGG	allele 1: △72
31	Male	GTTCCC---CTTCATGGG	allele 1: △72
32	Female	GTTCCCCTTCAGTTCATCTCCCCGGGGTGGAT//CAGTGCTTTGGCAACTTCTCCATGGG	allele 1: +10, +3
33	Female	GTTCCCCTTCAGTTCATCTCCCCGGGGTGGAT//CAGTGCTTTGGCAACTTCTCCATGGG	allele 1: +10, +3
34	Female	GTTCCC---CTTCATGGG	allele 1: △72
35	Female	GTTCCC---CTTCATGGG	allele 1: △72
36	Female	GTTCCCCTTCAGTTCATCTCCCCGGGGTGGAT//CAGTGCTTTGGCAACTTCTCCATGGG	allele 1: +10, +3

Note: Underlines mark target sites. Red text indicates base insertion; yellow highlighting indicates base mutation; (-) indicates base deletion. (+) denotes the number of inserted bases; (△) denotes the number of deleted bases; (→) represents the mutant base.
